# The Cognitive Functions of Youth With Obesity: A Meta‐Analysis

**DOI:** 10.1002/brb3.71884

**Published:** 2026-11-01

**Authors:** Yi‐Chun Lin, Chia‐Chia Chang, Yu‐Tzu Chang, Wei‐Lin Liu, Yu‐Chi Liao, Ro‐Ting Lin

**Affiliations:** ^1^ Department of Chinese Medicine China Medical University Hospital Taichung Taiwan; ^2^ Department of Psychology Asia University Taichung Taiwan; ^3^ School of Post Baccalaureate Chinese Medicine China Medical University Taichung Taiwan; ^4^ Division of Pediatric Neurology China Medical University Children's Hospital Taichung Taiwan; ^5^ Department of Physical Medicine and Rehabilitation Cathay General Hospital Taipei Taiwan; ^6^ Center for Prevention and Treatment of Internet Addiction Asia University Taichung Taiwan; ^7^ Clinical Psychology Center Asia University Hospital Taichung Taiwan; ^8^ Department of Occupational Safety and Health College of Public Health China Medical University Taichung Taiwan

**Keywords:** cognitive function, intelligence quotient, meta‐analysis, obesity, youth

## Abstract

**Background:**

This meta‐analysis examined the associations between obesity and performance across seven cognitive domains (inhibition, cognitive flexibility, working memory, verbal fluency, planning, attention, and intelligence quotient [IQ]) in youth under 18 years. Developmental stage (school‐aged children vs. adolescents) and sample sex composition examined as moderators. Evidence on executive function in youth with comorbid obesity and attention‐deficit/hyperactivity disorder (ADHD) was synthesized qualitatively.

**Methods:**

Following PRISMA guidelines, 22 studies comparing youth with obesity to normal‐weight peers were included. Effect sizes (Hedges’ *g*) were pooled using a random‐effects model. Subgroup analyses compared school‐aged children and adolescents; meta‐regression examined age and proportion of female participants as study‐level moderators.

**Results:**

Youth with obesity performed significantly worse than normal‐weight peers on inhibition (*g* ^+^ = −0.193), cognitive flexibility (*g* ^+^ = −0.499), working memory (*g* ^+^ = −0.652), verbal fluency (*g* ^+^ = −0.420), attention (*g* ^+^ = −0.526), and IQ (*g* ^+^ = −0.288); planning could not be estimated. School‐aged children showed deficits on inhibition, working memory, and attention; adolescents on working memory and attention only. Between‐subgroup tests were non‐significant for all domains (*p* ≥ 0.26), so developmental differences should be interpreted with caution. Meta‐regression showed that a higher proportion of female participants in a study sample, a study‐level characteristic rather than an individual‐level effect, was associated with larger working memory deficits (*p* = 0.002); age did not moderate any domain. Two ADHD‐comorbidity studies received qualitative synthesis only.

**Discussion:**

Obesity is associated with poorer cognitive performance in youth across several domains, with sex composition of the study sample emerging as a moderator of the working memory finding; differences between school‐aged children and adolescents were not significant and should be interpreted cautiously. Substantial heterogeneity, predominantly observational designs, inconsistent BMI criteria, and limited ADHD‐stratified data constrain causal inference. Future studies should standardize weight definitions, report age‐ and ADHD‐stratified data, and consider bidirectional pathways.

## Graphical abstract

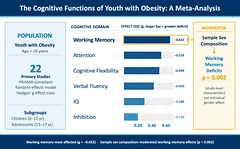

## 1 Introduction

Childhood and adolescent obesity has become an increasingly important global health concern. According to the World Health Organization, more than 390 million children and adolescents aged 5–19 years were overweight in 2022, and within this age group the prevalence of overweight increased from 8% in 1990 to 20% in 2022, while obesity rose from 2% to 8% over the same period (World Health Organization 2024). Overweight and obesity among young people have increased across both developed and developing countries (Ng et al. 2014), and are associated with elevated rates of hypertension, flatfoot, anxiety, and depression in children and adolescents (Zhang et al. 2024). Beyond these physical and psychological consequences, obesity may also be related to how children and adolescents think, regulate their behavior, and control their impulses. Executive functions such as inhibition, working memory, and cognitive flexibility support learning, classroom behavior, decision‐making, and everyday self‐control, and are considered foundational to healthy development (Diamond 2013); attention follows a partly distinct developmental course governed by alerting, orienting, and executive‐attention networks (Posner and Petersen 1990), and general intellectual ability (IQ) draws on this same prefrontal‐dependent architecture while also indexing broader academic and occupational outcomes (Nisbett et al. 2012). Several distinct mechanisms could plausibly link obesity to weaker performance across these domains: systemic low‐grade inflammation associated with excess adiposity (Samoilova et al. 2024), sleep‐disordered breathing and other sleep disturbances common in pediatric obesity (Pearce, Mackey, et al. 2018), structural and functional differences in prefrontal and reward‐related circuitry (Moreno‐López et al. 2012; Ronan et al. 2020), shared genetic liability affecting dopaminergic reward and self‐regulatory systems (Herzog et al. 2024), and shared environmental disadvantage, such as socioeconomic deprivation that affects both diet and cognitive stimulation (Li et al. 2023). Because prefrontal‐dependent cognitive systems are still maturing across childhood and adolescence, this developmental window is also when obesity‐prevention and cognitive‐support interventions may have the greatest long‐term benefit—the central stake motivating the present synthesis.

Despite this rationale, the empirical literature linking obesity to cognitive performance in youth has already been synthesized several times, with converging but incomplete conclusions. Liang et al. (2014), Reinert et al. (2013), Yang et al. (2018), and Favieri et al. (2019) each report small‐to‐moderate obesity‐related deficits concentrated in inhibition and working memory, alongside considerable inconsistency across primary studies and domains; of these, only Yang et al. (2018) conducted a formal meta‐analysis, while the others synthesized the evidence narratively. Most recently, Ásgeirsdóttir et al. (2026) conducted a preregistered meta‐analysis restricted to adolescents with obesity and found a small, domain‐general executive‐function deficit (*g* = −0.314) that did not differ significantly across specific domains, with age, BMI range, and the proportion of female participants in each sample moderating effect size. These reviews leave gaps that motivate the present synthesis: most combine overweight and obesity into a single category, obscuring any dose‐related pattern; none directly contrasts school‐aged children and adolescents within a single set of analyses; and, with the partial exception of Ásgeirsdóttir et al. (2026), none extends beyond core executive‐function domains to attention and IQ. Primary studies further illustrate why pooling, rather than additional narrative review, is needed: significant deficits associated with obesity have been reported for inhibition (Blanco‐Gómez et al. 2015; Kamijo et al. 2014; Kittel et al. 2017), cognitive flexibility (Alatorre‐Cruz et al. 2021; Blanco‐Gómez et al. 2015; Bozkurt et al. 2017; Maayan et al. 2011; Özer et al. 2024; Yau et al. 2014), working memory (Alatorre‐Cruz et al. 2021; Bozkurt et al. 2017; Kamijo, Khan, et al. 2012; Maayan et al. 2011; Özer et al. 2024), verbal fluency (Maayan et al. 2011), planning (Qavam et al. 2015), attention (Bozkurt et al. 2017; Chojnacki et al. 2018; Fields et al. 2013; Kittel et al. 2017; Logan et al. 2021; Maayan et al. 2011; Meo et al. 2019; Özer et al. 2024; Parisi et al. 2010), and IQ (Krombholz 2013; Maayan et al. 2011; Pearce, Mackey, et al. 2018)—while other studies of the very same domains report no significant group differences: inhibition (Kamijo, Pontifex, et al. 2012), cognitive flexibility (Meo et al. 2019), working memory (Bauer and Manning 2016; Goldschmidt et al. 2018), planning (Qavam et al. 2015), attention (Li et al. 2008; Moss et al. 2023), and IQ (Akın et al. 2017; Bozkurt et al. 2017; Fields et al. 2013; Kamijo, Khan, et al. 2012; Logan et al. 2021; Parisi et al. 2010; Yau et al. 2014). This pattern—genuine disagreement across studies rather than a simple accumulation of confirmatory evidence—is the hallmark of a question that a systematic quantitative synthesis is needed to resolve: only by pooling effect sizes across studies can the field distinguish true variability in the obesity–cognition association from sampling variability in individual, often small, studies.

We argue that this inconsistency is unlikely to be resolved by an overall pooled estimate alone, because there is good theoretical reason to expect the obesity–cognition association to vary systematically rather than to be uniform across youth. First, executive functions supported by the prefrontal cortex mature at markedly different rates across childhood and adolescence, and this maturation itself differs by sex, with prefrontal‐dependent networks—including those supporting working memory—tending to mature earlier in girls than in boys (Gur and Gur 2016; Lenroot and Giedd 2010). If obesity's association with cognition operates partly through, or interacts with, these maturational processes, the strength and pattern of obesity‐related cognitive differences should differ by developmental stage and plausibly by the sex composition of a given sample—predictions we test directly below using subgroup and moderator analyses. Second, ADHD offers a theoretically important test case for these mechanisms, because ADHD and obesity share overlapping neuropsychological correlates (inhibition, attention regulation, impulsivity, and working memory) that plausibly influence eating regulation and physical activity. Evidence directly linking obesity to executive functioning within ADHD populations is itself mixed: some studies report that higher body weight is associated with weaker inhibitory control and greater impulsivity among children and adolescents with ADHD (Graziano et al. 2012; Racicka‐Pawlukiewicz et al. 2021), while others find no such association (Hanć et al. 2018), and studies of specific executive domains in youth with both obesity and ADHD similarly disagree (Choudhry et al. 2013; Parhoon et al. 2023). This makes ADHD status a theoretically motivated candidate moderator of the obesity–cognition association. However, as described in the Methods, the literature search identified too few studies of children and adolescents with obesity that also report ADHD status to support a quantitative moderator analysis. We therefore treat ADHD‐related findings as a qualitative complement to the quantitative synthesis rather than as one of the moderators formally tested (see Section 3.2).

The pediatric meta‐analysis most directly comparable to the present one (Pearce, Leonhardt, et al. 2018) synthesized executive and reward‐related functioning in youth with obesity, but it did not examine attention or IQ, did not separate overweight from obesity, and did not contrast developmental stages; the present review extends that work on all three counts and, unlike Ásgeirsdóttir et al. (2026), includes school‐aged children alongside adolescents.

Based on the argument above, this meta‐analysis aimed to: (1) test whether obesity is associated with poorer performance across executive‐function domains (inhibition, cognitive flexibility, working memory, decision‐making, verbal fluency, planning) and broader neuropsychological outcomes (attention, IQ) in youth, predicting worse performance among youth with obesity consistent with prior meta‐analytic evidence (Yang et al. 2018; Ásgeirsdóttir et al. 2026); (2) test whether these associations differ by developmental stage and by the sex composition of study samples, predicting different patterns of association given the sex differences in the timing of prefrontal maturation noted above (Gur and Gur 2016; Lenroot and Giedd 2010); and (3) qualitatively synthesize the available evidence on obesity and cognition among youth with comorbid obesity and ADHD, given the theoretical but empirically underdetermined overlap between these conditions, without conducting a quantitative ADHD‐moderator analysis because too few directly comparable studies were available.

## 2 Methods

This systematic review and meta‐analysis was not prospectively registered in a registry such as PROSPERO. This review followed the Preferred Reporting Items for Systematic Reviews and Meta‐Analyses (PRISMA) reporting guideline to structure and document the review process (Table S1). Adherence to PRISMA is a reporting standard and does not, by itself, substitute for prospective registration; the absence of registration is acknowledged as a limitation of this review (see Section 4.7).

### 2.1 Study Selection and Inclusion/Exclusion Criteria

#### 2.1.1 PECO

The study's research question was structured according to the PECO framework as follows: (1) population: infant, preschool child, children, and adolescent; (2) index condition (hereafter “E,” retaining the PECO label, but denoting group membership rather than a temporal or causal exposure, consistent with the predominantly cross‐sectional design of the included literature): group with obesity; (3) comparator: normal weight group; (4) outcome: executive function, attention, or IQ test score were assessed, diagnosis with or without ADHD. We followed this PECO framework to generate our search terms for three databases: Medline, PubMed, and Embase (Table S2).

#### 2.1.2 Literature Review

A topic search was conducted in Medline, PubMed, and Embase to identify studies published up to December 17, 2024. For each database, search terms combining three concept blocks—(1) obesity/overweight, (2) executive function, attention, IQ, or ADHD, and (3) infant/child/adolescent/youth—were connected with Boolean AND/OR operators and adapted to each database's controlled vocabulary (e.g., MeSH, Emtree) and syntax; the complete, database‐specific search strings are provided in Table S2. The search retrieved 7837 records from Medline, 1065 from PubMed, and 657 from Embase. All records were imported into EndNote 21 for reference management and screening. Titles and abstracts were screened first, followed by full‐text review for articles that appeared potentially eligible based on their title or abstract. The detailed study selection process is illustrated in Figure 1. Two reviewers independently screened all titles, abstracts, and full‐text articles against the predefined eligibility criteria (Table 1). Disagreements were resolved through discussion until consensus was reached, and a third senior reviewer was consulted when discrepancies persisted. Data from each eligible study were extracted using a standardized, piloted data‐extraction form that recorded study characteristics, country, design, sample sizes, participant age and sex composition, weight‐classification criteria, the cognitive tasks administered, and the summary statistics (means, standard deviations, and sample sizes) required for effect‐size computation.

**FIGURE 1:**
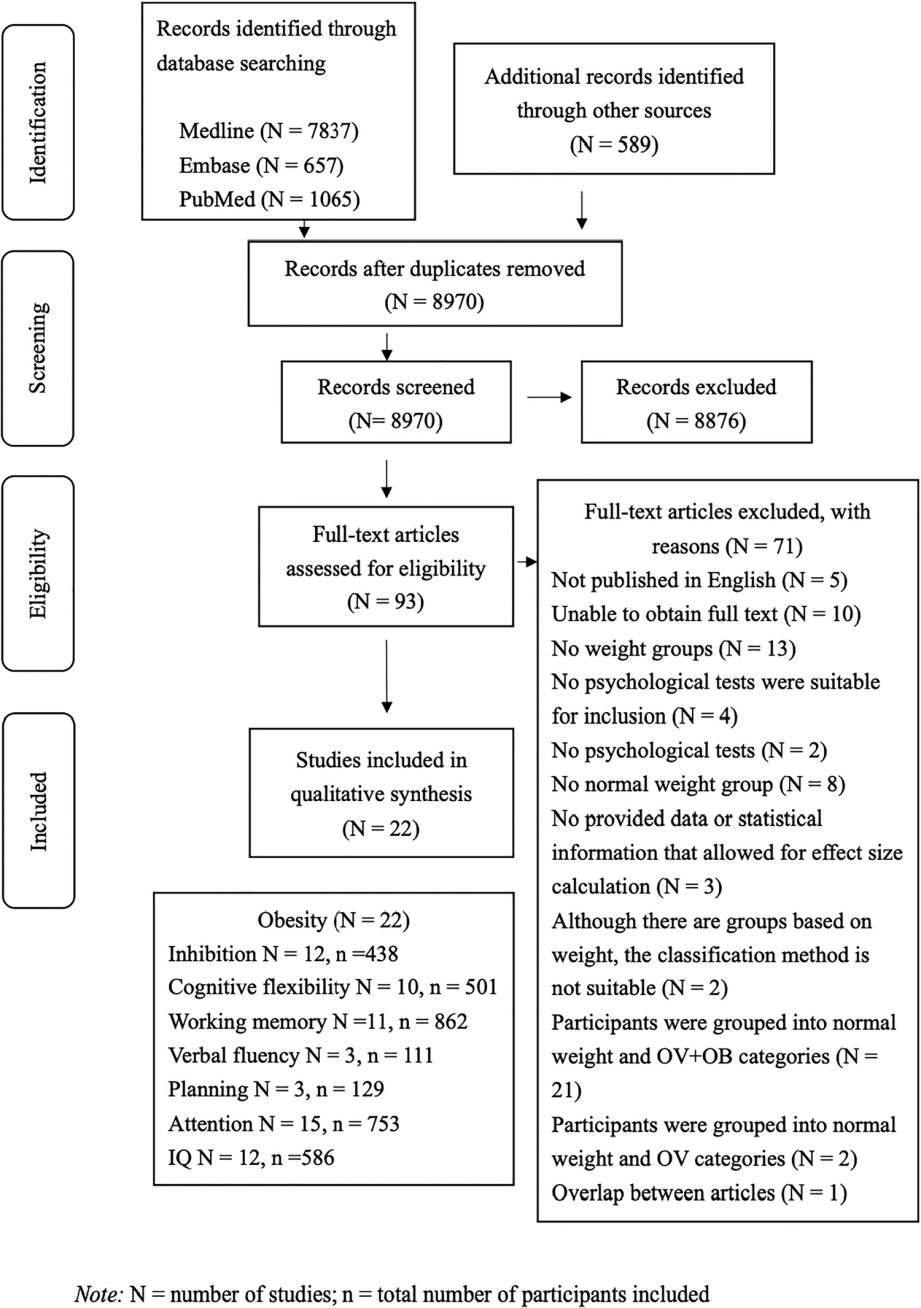
PRISMA flow diagram illustrating the literature search, screening, eligibility assessment, and final inclusion process. The diagram shows the number of records identified, duplicates removed, studies screened, and studies included in the meta‐analysis.

**TABLE 1:** Study eligibility criteria.

Inclusion criteria	Exclusion criteria
Examined human participants. Compared at least one group of individuals with obesity to a normal‐weight control group, with or without ADHD. Used at least one task known or shown to depend upon or assess executive function, attention, or IQ. Provided data or statistical information that allowed for effect size calculation.	Included participants with a known mental illness (e.g., depression, schizophrenia, bipolar disorder, binge eating disorder). Included participants with a known physiological illness (e.g., hypertension, diabetes). Did not distinguish between overweight and obesity but instead combined them into a single group (OV+OB). Included participants whose mean age was 18 years or older—that is, only studies in which the mean age of participants was under 18 years were eligible. Included participants who had undergone bariatric surgery. Were not published in English or Chinese.

Studies were eligible if they met all inclusion criteria and none of the exclusion criteria below (Table 1).

#### 2.1.3 Selected Studies

The analysis included data from 22 studies comparing youth with obesity and normal‐weight participants. Across these studies, the most commonly assessed domains were attention (15 studies), inhibition (12 studies), IQ (12 studies), working memory (11 studies), and cognitive flexibility (10 studies). Fewer studies examined verbal fluency and planning, with three studies available for each domain. For subgroup analyses, studies were further classified into two developmental‐stage groups on the basis of the age range and developmental stage of each sample as reported by the original authors (Table 2): school‐aged children, comprising samples aged 6–12 years (sample mean age < 12 years; *k* = 9: Akın et al. 2017; Alatorre‐Cruz et al. 2021; Blanco‐Gómez et al. 2015; Chojnacki et al. 2018; Choudhry et al. 2013; Kamijo, Khan, et al. 2012; Kamijo et al. 2014; Logan et al. 2021; Parhoon et al. 2023), and adolescents, comprising samples aged 13–17 years (*k* = 6: Fields et al. 2013; Kittel et al. 2017; Maayan et al. 2011; Mayer et al. 2022; Meo et al. 2019; Yau et al. 2014). The boundary between 12 and 13 years was chosen because it approximates the transition from middle childhood to adolescence, marked by pubertal onset and by the beginning of the protracted adolescent phase of prefrontal maturation (Lenroot and Giedd 2010), and because it coincides with the child/adolescent distinction used by the original studies themselves. Studies whose samples spanned both stages without reporting age‐stratified data (Bozkurt et al. 2017; Li et al. 2008; Özer et al. 2024; Parisi et al. 2010; Pearce, Mackey, et al. 2018) were retained in the overall analyses only. The single study of preschool children (Krombholz 2013; mean age 5.5 years) was likewise retained only in the overall analyses, because one study cannot support a separate preschool subgroup and pooling it with school‐aged samples would have blurred the developmental contrast. Qavam et al. (2015), an adolescent sample, contributed only to the planning domain, for which no subgroup analysis was possible. The study‐by‐study allocation is shown in Table 3.

**TABLE 2:** A comprehensive overview of the 22 studies included and analyzed in the meta‐analysis (normal weight vs. obesity).

Study	Country	Subject (sample size)	Female (%)	Development range	Mean age (years) or age range (years)	BMI definition	Mean BMI (kg/m^2^) or percentile (%)	Design	Control
Akın et al. (2017)	Türkiye	NW (42) OB (73)	50 % 47.9 %	Child	11.5 11.8	BMI >95th	N. R. N. R.	Cross‐sectional	Age Gender
Alatorre‐Cruz et al. (2021)	USA	NW (29) OB (27)	62.1 % 62.96 %	Child	9.6 9.65	BMI >95th	57.3% 96.9%	Cross‐sectional	Age Gender
Blanco‐Gómez et al. (2015)	Spain	NW (90) OB (36)	40 % 50 %	Child	6‐10 6‐10	BMI >97th	N. R. N. R.	Cross‐sectional	Age Gender
Bozkurt et al. (2017)	Türkiye	NW (55) OB (92)	54.5% 56.5%	Child Adolescent	11.9 11.85	BMI >95th	21.07 kg/m^2^ 29.73 kg/m^2^	Cross‐sectional	Age Gender
Chojnacki et al. (2018)	USA	NW (131) OB (53)	N. R. N. R.	Child	7‐9 7‐9	BMI >95th	N. R. N. R.	Cross‐sectional	Age
Choudhry et al. (2013)	Canada	NW (168) OB (59)	25% 22%	Child	9.01 9.6	BMI >97th	N. R. N. R.	Cross‐sectional	Age Gender
Fields et al. (2013)	USA	NW (20) OB (21)	60% 52.4%	Adolescent	15 14.86	BMI >95th	20.41 kg/m^2^ 34.39 kg/m^2^	Cross‐sectional	Age
Kamijo et al. (2012)	USA	NW (70) OB (30)	48.6% 56.7%	Child	8.9 9	BMI >95th	16.5 kg/m^2^ 26.1 kg/m^2^	Cross‐sectional	Age Gender
Kamijo et al. (2014)	USA	NW (37) OB (37)	54.1% 54.1%	Child	8.8 8.9	BMI >95th	56.8% 98%	Cross‐sectional	Age Gender
Kittel et al. (2017)	Germany	NW (22) OB (22)	81.8% 81.8%	Adolescent	15.23 14.82	BMI >97th	58.91% 98.91%	Cross‐sectional	Age Gender
Krombholz (2013)	Germany	NW (1292) OB (70)	N. R. N. R.	Preschool child	5.41 5.51	BMI >97th	15.5 kg/m^2^ 20.9 kg/m^2^	Cross‐sectional	Age
Li et al. (2008)	USA	NW (1746) OB (360)	N. R. N. R.	Child Adolescent	8‐16 8‐16	BMI >95th	N. R. N. R.	Cross‐sectional	Age Gender
Logan et al. (2021)	USA	NW (47) OB (47)	61.7% 61.7%	Child	8.82 8.82	BMI >95th	N. R. N. R.	Randomized controlled Trial	Age Gender
Maayan et al. (2011)	USA	NW (37) OB (54)	56.8% 62.9%	Adolescent	17.32 17.5	BMI >95th	21.67 kg/m^2^ 39.86 kg/m^2^	Cross‐sectional	Age Gender
Mayer et al. (2022)	Argentina	NW (363) OB (54)	N. R. N. R.	Adolescent	13‐14 13‐14	IOTF	N. R. N. R.	Prospective cohort	Age Gender
Meo et al. (2019)	Saudi Arabia	NW (92) OB (118)	0% 0%	Adolescent	14 13.85	BMI 30‐34.99 kg/m^2^	N. R. N. R.	Cross‐sectional	Age Gender
Özer et al. (2024)	Türkiye	NW (33) OB (33)	54.55% 54.55%	Child Adolescent	11.89 11.58	BMI >95th	N. R. N. R	Case‐ control	Age Gender
Parhoon et al. (2023)	Iran	NW (30) OB (30)	46.7% 50%	Child	10.77 10.37	BMI >97.72th	N. R. N. R	Case‐ control	Age Gender
Parisi et al. (2010)	Italy	NW (188) OB (51)	N. R. N. R.	Child	6‐13 6‐13	BMI >95th	17.3kg/m^2^ 23.6 kg/m^2^	Cross‐sectional	Age
Pearce et al. (2018)	USA	NW (30) OB (29)	N. R. N. R.	Child Adolescent	11.9 11.4	BMI >95th	60.7% 98.5%	Cross‐sectional	Age
Qavam et al. (2015)	Iran	NW (40) OB (40)	0% 0%	Adolescent	16.1 16.03	BMI >95th	N. R. N. R.	Cross‐sectional	Age Gender
Yau et al. (2014)	USA	NW (30) OB (30)	63.3% 56.7%	Adolescent	17.22 17.64	BMI >95th	21.12 kg/m^2^ 35.47 kg/m^2^	Cross‐sectional	Age Gender

*Note*: N.R. = not reported; NW = normal weight group; OB = obesity group. This table represents various global regions including the United States, Türkiye, Germany, and Iran. It details the sample sizes (*n*) for both normal weight (NW) and obese (OB) groups—ranging from small clinical samples (e.g., *n* = 20 per group) to large‐scale studies (e.g., *n* = 1746 for NW). The table also lists developmental stages (preschool, child, adolescent), mean ages, and specific BMI definitions used for classification (mostly >95th or >97th percentile). Most studies utilized a cross‐sectional design and matched controls based on age and gender.

**TABLE 3:** Classification of executive function domains and neuropsychological tasks.

Study	Subject (sample size)	Executive functions studied	Task(s) used	Analysis
Akın et al. (2017)	NW (42)	IQ	FSIQ	O + C
	OB (73)			
Alatorre‐Cruz et al. (2021)	NW (29) OB (27)	Inhibition Cognitive flexibility	D‐KEFS Color Word‐inhibition D‐KEFS TMT	O + C
		Working memory	WRAT‐math computation	
		Verbal fluency	D‐KEFS Letter Fluency	
		Impulsivity	WCST‐perseverative errors	
Blanco‐Gómez et al. (2015)	NW (90) OB (36)	Inhibition Cognitive flexibility	FDT‐inhibition FDT‐flexibility	O + C
		Attention	SDMT	
Bozkurt et al. (2017)	NW (55) OB (92)	Cognitive flexibility Working memory Attention IQ	CNSVS‐cognitive flexibility CNSVS‐verbal memory CNSVS‐complex attention Stanford‐Binet IQ Test	O
Chojnacki et al. (2018)	NW (131) OB (53)	Inhibition Attention	Flanker Task I‐CVRT Flanker Task C‐CVRT	O + C
Choudhry et al. (2013)	NW (168) OB (59)	Inhibition Cognitive flexibility Working memory Planning Attention Impulsivity IQ	Stroop Test WCST Self‐Ordered Pointing Test Tower of London Task CPT‐omissions CPT‐commissions FSIQ	O + C
Fields et al. (2013)	NW (20) OB (21)	Inhibition Attention IQ	Go‐Stop Task CPT‐omissions Kaufman Brief Intelligence Test	O + A
Kamijo, Khan et al. (2012)	NW (70) OB (30)	Inhibition Working memory IQ	Go/No‐Go WRAT‐arithmetic Kaufman Brief Intelligence Test	O + C
Kamijo et al. (2014)	NW (37) OB (37)	Attention	Flanker Task C‐RT	O + C
Kittel et al. (2017)	NW (22) OB (22)	Inhibition Cognitive flexibility Attention	CWIT CTMT‐complex sequencing CTMT‐simple sequencing	O + A
Krombholz (2013)	NW (1292) OB (70)	Attention IQ	CWIT Concentration Intelligence	O
Li et al. (2008)	NW (1746) OB (360)	Working memory	Arithmetic Test	O
Logan et al. (2021)	NW (47) OB (47)	Inhibition Attention IQ	Modified Flanker Task I‐RA Modified Flanker Task C‐RA Kaufman Brief Intelligence Test	O + C
Maayan et al. (2011)	NW (37) OB (54)	Cognitive flexibility Working memory Verbal fluency Attention IQ	TMT B WRAML‐WMIS COWAT TMT A FSIQ	O + A
Mayer et al. (2022)	NW (363) OB (54)	Inhibition	Go/No‐Go	O + A
Meo et al. (2019)	NW (92) OB (118)	Cognitive flexibility Working memory Attention	IED SRM AST	O + A
Özer et al. (2024)	NW (33) OB (33)	Cognitive flexibility Working memory Attention	TMT B CNSVS‐cognitive flexibility CNSVS‐composite memory CNSVS‐complex attention	O
Parhoon et al. (2023)	NW (30) OB (30)	Inhibition Cognitive flexibility Working memory Planning Attention Impulsivity IQ	Victoria Stroop Test WCST WISC V‐Digit Span scaled score Tower of London Task Dot‐Probe Task WCST‐perseverative errors FSIQ	O + C
Parisi et al. (2010)	NW (188) OB (51)	Attention IQ	WISC‐R Kauffman Factors‐attention ability FSIQ	O
Pearce et al. (2018)	NW (30) OB (29)	Inhibition Working memory IQ	Stop‐Signal Task SSRT N‐Back‐2‐Back accuracy FSIQ	O
Qavam et al. (2015)	NW (40) OB (40)	Planning	Tower of London Task	O
Yau et al. (2014)	NW (30) OB (30)	Inhibition Cognitive flexibility Working memory Verbal fluency Attention Impulsivity IQ	Stroop Test TMT B WRAML‐WMIS COWAT Digit Symbol Substitution Test WCST‐perseverative errors FSIQ	O + A

*Note*: Analysis = analyses to which the study contributed: O = overall analyses only; O + C = overall and school‐aged‐children (612 years) subgroup; O + A = overall and adolescent (1317 years) subgroup. NW = normal weight group; OB = obesity group; TMT A = Trail Making Test A; TMT B = Trail Making Test B; WCST = Wisconsin Card Sorting Test; WCST‐perseverative errors = Wisconsin Card Sorting Test perseverative errors; CWIT = Color‐Word Interference Test; CTMT‐ complex sequencing = Comprehensive Trail Making Test complex sequencing; CTMT‐simple sequencing = Comprehensive Trail Making Test simple sequencing; FDT‐inhibition = Five Digit Test inhibition; FDT‐flexibility = Five Digit Test flexibility; SDMT = Symbol Digit Modalities Test; CNSVS‐cognitive flexibility = Central Nervous System Vital Signs cognitive flexibility; CNSVS‐verbal memory = Central Nervous System Vital Signs verbal memory; CNSVS‐complex attention = Central Nervous System Vital Signs complex attention; CNSVS‐composite memory = Central Nervous System Vital Signs composite memory; Flanker Task I‐CVRT = Flanker Task Incongruent coefficient of variation of reaction time; Flanker Task C‐CVRT = Flanker Task Congruent coefficient of variation of reaction time; Flanker Task C‐RT = Flanker Task Compatible reaction time; CPT‐commissions = Conner's Continuous Performance Test commissions; CPT‐omissions = Conner's Continuous Performance Test omissions; WRAT‐arithmetic = Wide Range Achievement Test arithmetic; WRAT‐math computation = Wide Range Achievement Test math computation; IED = Intra‐Extra Dimensional Set Shift; SRM = Spatial Recognition Memory; AST = Attention Switching Task; WRAML‐ WMIS = Wide Range Assessment of Memory and Learning working memory index score; COWAT = Controlled Oral Word Association Test; WISC V‐Digit Span scaled score = Wechsler Intelligence Scale for Children V Digit Span scaled score; D‐KEFS Color Word‐inhibition = DelisKaplan Executive Function System Color Word inhibition; D‐KEFS TMT = DelisKaplan Executive Function System.

Trail Making Test; D‐KEFS Letter Fluency = DelisKaplan Executive Function System Letter Fluency; Modified Flanker Task I‐RA = Modified Flanker Task Incongruent response accuracy; Modified Flanker Task C‐RA = Modified Flanker Task Congruent response accuracy; WISC‐R Kauffman Factors‐attention ability = Wechsler Intelligence Scale for Children‐Revised Kauffman Factors attention ability; Stop‐Signal Task SSRT = Stop‐Signal Task stop‐signal reaction time.

### 2.2 Coding of Variables

Cognitive outcomes, including executive function and IQ, were classified into domains using prior theoretical and empirical frameworks and the descriptions provided in the original studies. Because some neuropsychological tests draw on more than one cognitive process—for example, the Stroop task is commonly used to index inhibitory control but also engages attentional processes—each task was assigned to the single cognitive domain it is most commonly used to index in the developmental executive‐function literature, consistent with established taxonomies of executive function (e.g., Miyake et al. 2000), and following the primary construct identified by the authors of the original study. When a task's classification could plausibly fall under more than one domain, priority was given to the domain the original study explicitly identified as its primary outcome. The complete task‐to‐domain coding is shown in Table 3, which served as the reference for the meta‐analysis.

Mean age and percentage of female participants in the obesity group were coded as continuous variables. Obesity was operationalized using the BMI criteria reported by each study. Most studies defined obesity as a BMI at or above the 95th percentile for age and sex, although some used the 97th percentile, absolute BMI cut‐offs, or IOTF and WHO reference standards (Table 2). Studies were excluded when overweight and obesity were combined and could not be analyzed separately.

### 2.3 Quality Assessment Using the Newcastle–Ottawa Scale (NOS)

The Newcastle–Ottawa Scale (NOS) is widely used in systematic reviews and meta‐analyses to assess the quality of non‐randomized studies, such as cohort and case‐control studies. It evaluates studies across three main domains: (1) selection: representativeness of the study population, selection of the exposed and non‐exposed cohorts, and ascertainment of exposure; (2) comparability: control of key confounding variables between study groups; (3) outcome/exposure: assessment of outcomes and adequacy of follow‐up (or, in the case of cross‐sectional studies, the accuracy of exposure and outcome measurements) (Wells et al. 2000). Although initially designed for cohort and case‐control studies, the NOS framework can be flexibly applied or adapted to evaluate cross‐sectional studies, particularly in terms of assessing selection bias and controlling confounders. It employs a star‐rating system to quantitatively assess the methodological quality of individual studies, with a maximum score of 9 stars: up to four stars for selection, two stars for comparability, and three stars for outcome/exposure. The detailed scores of each study are illustrated in Table S3. A formal quality and risk‐of‐bias assessment was therefore conducted for every included study using the NOS. No study was excluded solely on the basis of its quality rating; instead, NOS scores were taken into account when interpreting the robustness of the pooled findings.

### 2.4 Statistical Analyses

All statistical analyses were conducted using Comprehensive Meta‐Analysis software (Version 3.0; Biostat, Englewood, NJ, USA). Hedges’ *g* was used as the effect size measure because it provides a bias‐corrected standardized mean difference and is suitable when included studies have relatively small sample sizes. Given the expected variation in cognitive measures, participant characteristics, and study designs, pooled effects were estimated using a random‐effects model. Effect sizes were calculated from means, standard deviations, and sample sizes according to Borenstein et al. (2021). Between‐study heterogeneity was quantified with the *I* ^2^ statistic (Higgins et al. 2003). The magnitude of effects was interpreted using conventional thresholds: small (*g* = 0.2), medium (*g* = 0.5), and large (*g* = 0.8).

Moderator analyses were conducted using meta‐regression to examine whether mean age and the percentage of female participants were associated with differences in obesity‐related cognitive outcomes. These moderators were selected in advance because previous theoretical and empirical work suggests that cognitive outcomes may vary by developmental stage and sex composition. Percentage of female participants is a study‐level aggregate describing the sex composition of each sample, not an individual‐level measure; any moderating association therefore describes differences between study samples that vary in their proportion of female participants and does not support inferences about individual girls versus boys. Two covariates (mean age and percentage female) were tested per outcome domain without adjustment for multiple comparisons, so moderator findings are best treated as exploratory rather than confirmatory (see Discussion). Subgroup analyses were also conducted to compare findings between school‐aged children (6–12 years) and adolescents (13–17 years); because within‐subgroup significance can diverge between two subgroups without the subgroups differing significantly from one another, a formal test of subgroup differences was additionally conducted for domains with pooled estimates in both subgroups (see Section 3). Publication bias was assessed using funnel plot asymmetry, Egger's test (Egger et al. 1997), and Begg's test (Begg and Mazumdar 1994). In all subsequent analyses, a negative effect size indicates that the group with obesity performed worse than the normal‐weight control group, whereas a positive effect size reflects better performance in the group with obesity. As the outcome variable in these analyses is the standardized mean difference between groups (i.e., the effect size), a significant moderator implies that the effect size varies as a function of that moderator variable. Meta‐regression analyses were conducted only for outcomes with at least 10 studies per covariate, in accordance with recommended guidelines (Borenstein et al. 2021). Because Egger's and Begg's tests have limited statistical power when the number of studies is small, results for domains with fewer than 10 studies (*k* < 10) are interpreted with caution in Section 3 and flagged accordingly (Sterne et al. 2011).

## 3 Results

### 3.1 Study Characteristics

The final sample consisted of 22 studies that compared cognitive functioning (executive function, attention, and IQ) between youth with obesity and normal weight individuals (Tables 2 and 3) (Akın et al. 2017; Alatorre‐Cruz et al. 2021; Blanco‐Gómez et al. 2015; Bozkurt et al. 2017; Chojnacki et al. 2018; Choudhry et al. 2013; Fields et al. 2013; Kamijo, Khan, et al. 2012; Kamijo et al. 2014; Kittel et al. 2017; Krombholz 2013; Li et al. 2008; Logan et al. 2021; Maayan et al. 2011; Mayer et al. 2022; Meo et al. 2019; Özer et al. 2024; Parhoon et al. 2023; Parisi et al. 2010; Pearce, Mackey, et al. 2018; Qavam et al. 2015; Yau et al. 2014). However, 21 studies combined overweight and obesity into a single group (OV+OB) without reporting separate subgroup data and were therefore excluded from obesity‐specific analyses.

Across 22 studies, Attention was assessed most often, with 15 studies including 753 participants with obesity. Inhibition and IQ were each examined in 12 studies, involving 438 and 586 participants with obesity, respectively. Working memory was assessed in 11 studies with 862 participants with obesity, while cognitive flexibility was examined in 10 studies with 501 participants. Evidence was more limited for verbal fluency and planning, with three studies available for each domain and 111 and 129 participants with obesity, respectively.

The included studies represented a broad range of geographic settings, including the United States, Türkiye, Germany, Spain, Italy, Canada, Iran, Argentina, India, and Saudi Arabia. Most used cross‐sectional designs, although some studies applied case‐control, prospective cohort, or randomized controlled designs (Table 2).

Sample sizes ranged from small clinical samples (*n* = 20–30 per group) to large population‐based cohorts (*n* > 1700). Most studies classified obesity using BMI percentile thresholds (≥ 95th or ≥ 97th) and matched the comparison groups on age and sex. Across studies, the general direction of effects was consistent, with youth with obesity tending to perform more poorly than their normal‐weight peers, although the magnitude of these differences varied by cognitive domain and developmental stage. The specific studies contributing to each cognitive domain, together with the corresponding tasks, are identified in Table 3, allowing readers to determine which studies informed each pooled estimate.

Table 3 also indicates, for each study, whether it contributed to the overall analyses only or also to the school‐aged‐children or adolescent subgroup analyses, so that the overall and subgroup *k* for each domain can be reconciled (see Section 2.1.3).

### 3.2 ADHD Status: Qualitative Synthesis

Two of the 22 included studies specifically compared cognitive performance in youth with obesity and comorbid ADHD (Choudhry et al. 2013; Parhoon et al. 2023). Because this is too few studies to support a quantitative moderator analysis, ADHD‐related findings are synthesized narratively here rather than pooled statistically, consistent with the approach outlined in the Introduction and Methods. Parhoon et al. (2023) reported that Iranian children with both ADHD and obesity performed worse than children with ADHD alone on measures of inhibition, cognitive flexibility, and working memory, suggesting that obesity may compound the executive dysfunction already associated with ADHD. In contrast, Choudhry et al. (2013) found no significant differences in inhibition, cognitive flexibility, working memory, or planning between children with ADHD with and without obesity. These two studies could not be reconciled quantitatively, and their divergent findings indicate that the relationship between obesity and cognition among youth with ADHD remains unresolved and represents an important gap for future primary research (see Section 4.7). To be precise about what was compared: both studies contrast youth with comorbid obesity and ADHD to youth with ADHD at normal weight, not youth with and without ADHD—every participant in both studies had ADHD, and obesity status was the between‐group comparison. These two studies also remain included in the overall and domain‐level quantitative analyses above, since the inclusion criteria (Table 1) did not require ADHD status to be reported; their data are therefore pooled with the other 20 studies at the level of the obesity‐versus‐normal‐weight comparison, even though the ADHD‐specific findings are treated qualitatively here rather than tested as a separate quantitative moderator. Finally, the database search strings (Table S2) were built around obesity and cognitive‐outcome terms and did not include ADHD‐specific search terms; the two ADHD studies above were identified because they also satisfied the general obesity and cognitive‐outcome criteria, not through a dedicated ADHD search arm. A review specifically targeting obesity–ADHD comorbidity would require its own search strategy and may identify additional eligible studies.

### 3.3 Obesity and Cognitive Function: Pooled Associations Across Domains

#### 3.3.1 Inhibition

Analysis of 12 studies showed a significant overall effect of obesity on inhibition (*g* ^+^ = –0.193, *p* = 0.049, 95% CI [–0.386, –0.001]; Figure 2A), indicating poorer inhibitory control in participants with obesity compared to normal‐weight peers. Moderate heterogeneity was observed (*I* ^2^ = 59.6%), reflecting some variability across studies. In the assessment of obesity (Figure 3A), no publication bias was detected by Egger's (*t*(10) = 0.137, *p* = 0.894) or Begg's test (*p* = 0.945). The proportion of female participants did not significantly moderate this effect (Table 4); mean age was not tested as a moderator for this domain.

**FIGURE 2:**
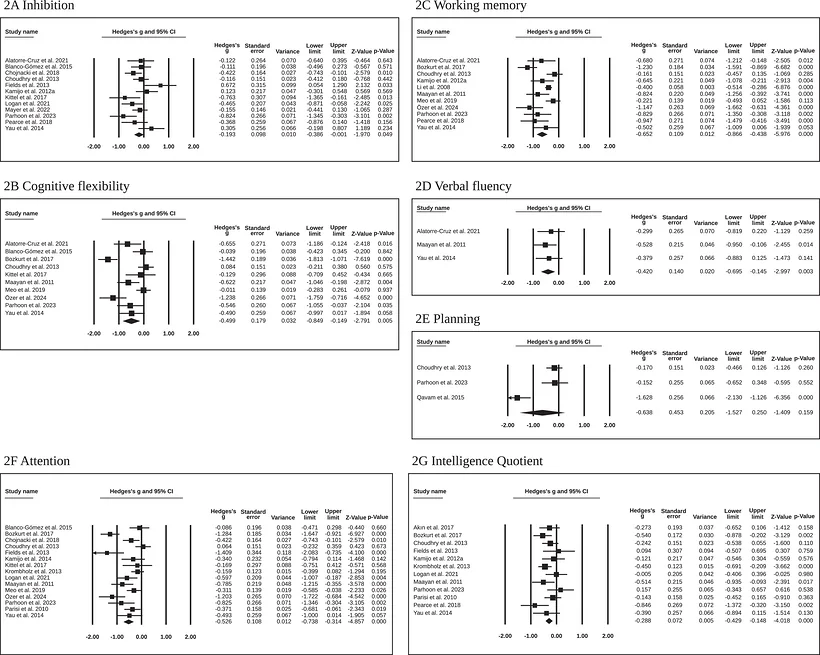
Forest plots displaying meta‐analytic effect sizes (Hedges’ *g* with 95% confidence intervals) for differences in cognitive performance between youth with obesity and normal‐weight controls (overall sample). Panels: (A) inhibition (*k* = 12, *n* = 438 with obesity); (B) cognitive flexibility (*k* = 10); (C) working memory (*k* = 11); (D) verbal fluency (*k* = 3); (E) planning (*k* = 3); (F) attention (*k* = 15); (G) IQ (*k* = 12). Negative effect sizes indicate poorer performance in the obesity group. The diamond represents the pooled effect size.

**FIGURE 3:**
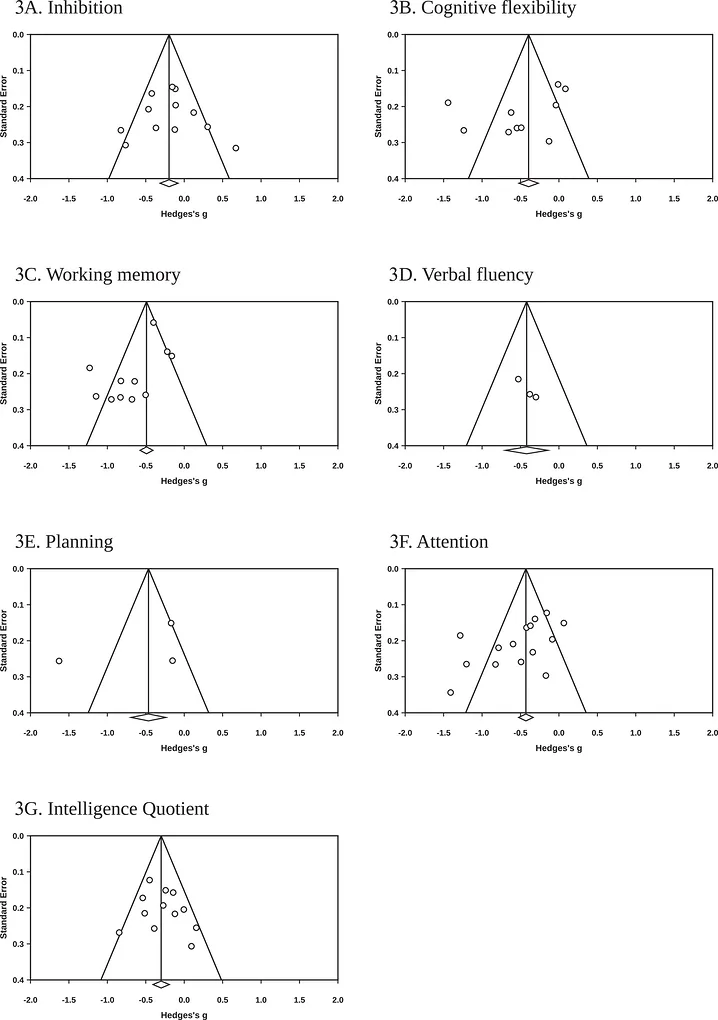
Funnel plots assessing publication bias for the overall sample (combined age groups) across executive function and neuropsychological domains. Panels: (A) inhibition; (B) cognitive flexibility; (C) working memory; (D) verbal fluency; (E) planning; (F) attention; (G) IQ. Each plot displays individual study effect sizes (Hedges’ *g*) against their standard errors; Egger's and Begg's test results are reported in Section 3.

**TABLE 4:** Meta‐regression analysis for potential moderators of executive function.

EF	Variable(s)	Lower limit	Upper limit	*Q*	df	*P*‐value
Inhibition	Percent female	−0.024	0.012	0.45	1	0.504
Working memory	Participant age	−0.097	0.093	0.00	1	0.971
	**Percent female**	**−0.021**	**−0.005**	**9.66**	**1**	**0.002**
Attention	Participant age	−0.113	0.032	1.20	1	0.274
	Percent female	−0.021	0.005	1.36	1	0.244
Intelligence quotient	Participant age	−0.054	0.036	0.15	1	0.695
	Percent female	−0.020	0.011	0.33	1	0.568

*Note*: This table presents the results of meta‐regression analyses used to determine if participant age or the percentage of females influenced the observed differences in EF. Key statistical metrics include the *Q*‐value (test of heterogeneity), degrees of freedom (df), and *p*‐value.

#### 3.3.2 Cognitive Flexibility

Analysis of 10 studies showed a significant overall effect of obesity on cognitive flexibility (*g* ^+^ = –0.499, *p* = 0.005, 95% CI [–0.849, –0.149]; Figure 2B), with participants with obesity performing worse than normal‐weight peers. High heterogeneity was observed (*I* ^2^ = 86.2%), suggesting variability across studies. No publication bias was detected by Egger's (*t*(8) = 1.488, *p* = 0.175) or Begg's test (*p* = 0.371; Figure 3B). Although 10 studies contributed to this domain, moderator analyses for age and sex were not conducted because the two developmental‐stage subgroups contained only four studies each, leaving too little between‐study variation in mean age and sex composition to model reliably.

#### 3.3.3 Working Memory

Analysis of 11 studies showed a significant overall effect of obesity on working memory (*g* ^+^ = –0.652, *p* < 0.0001, 95% CI [–0.866, –0.438]; Figure 2C), with participants with obesity performing worse than normal‐weight peers. Substantial heterogeneity was observed (*I* ^2^ = 75.6%), indicating variability across studies. No publication bias was detected (Egger's *t*(9) = 2.17, *p* = 0.058; Begg's *p* = 0.213; Figure 3C). Age did not moderate this effect, whereas the percentage of female participants showed a significant moderating influence (Table 4).

#### 3.3.4 Decision‐Making

There are insufficient studies to examine the differences in decision‐making between participants with obesity and normal‐weight participants.

#### 3.3.5 Verbal Fluency

Analysis of three studies revealed a significant overall effect of obesity on verbal fluency (*g* ^+^ = –0.420, *p* = 0.003, 95% CI [–0.695, –0.145]; Figure 2D), with no heterogeneity (*I* ^2^ = 0%). No publication bias was detected (Egger's *t*(1) = 5.496, *p* = 0.115; Begg's *p* = 0.296; Figure 3D). Due to limited data, moderator analyses were not conducted.

#### 3.3.6 Planning

Across three studies, planning showed a non‐significant effect (*g* ^+^ = –0.638, *p* = 0.159, 95% CI [–1.527, 0.250]; Figure 2E) and high heterogeneity (*I* ^2^ = 92.3%). No publication bias was detected (Egger's *t*(1) = 0.755, *p* = 0.588; Begg's *p* = 0.296; Figure 3E). Moderator analyses were not feasible due to limited data.

#### 3.3.7 Attention

Analysis of 15 studies showed a significant overall effect of obesity on attention (*g* ^+^ = –0.526, *p* < 0.0001, 95% CI [–0.738, –0.314]; Figure 2F). High heterogeneity was observed (*I* ^2^ = 78.1%). Egger's (*t*(13) = 2.407, *p* = 0.032) and Begg's tests (*p* = 0.038) indicated possible publication bias (Figure 3F). Neither age nor female proportion significantly moderated the effect (Table 4). To address the detected publication bias, we conducted a trim‐and‐fill analysis (Duval and Tweedie 2000). The *L*_0_ estimator imputed no missing studies (*k*_0_ = 0), and the adjusted pooled estimate was identical to the original (*g* ^+^ = −0.526, 95% CI [−0.738, −0.314]), suggesting that the funnel asymmetry reflects small‐study effects rather than selective non‐publication of null findings.

#### 3.3.8 Intelligence Quotient (IQ)

Analysis of 12 studies revealed a significant overall effect of obesity on IQ (*g* ^+^ = –0.288, *p* < 0.0001, 95% CI [–0.429, –0.148]; Figure 2G), with moderate heterogeneity (*I* ^2^ = 36.9%). No publication bias was detected (Egger's *t*(10) = 0.75, *p* = 0.470; Begg's *p* = 0.631; Figure 3G). Age and sex were not significant moderators (Table 4).

### 3.4 Subgroup Associations Between Obesity and Cognitive Function (School‐Aged Children, 6–12 Years)

#### 3.4.1 Inhibition

Analysis of seven studies showed a significant overall effect of obesity on inhibition (*g* ^+^ = –0.261, *p* = 0.013, 95% CI [–0.467, –0.055]; Figure 4A), with school‐aged children with obesity performing worse than normal‐weight peers. Moderate heterogeneity was observed (*I* ^2^ = 47.1%). No publication bias was detected (Egger's *t*(5) = 0.51, *p* = 0.632; Begg's *p* = 0.548; Figure 5A).

**FIGURE 4:**
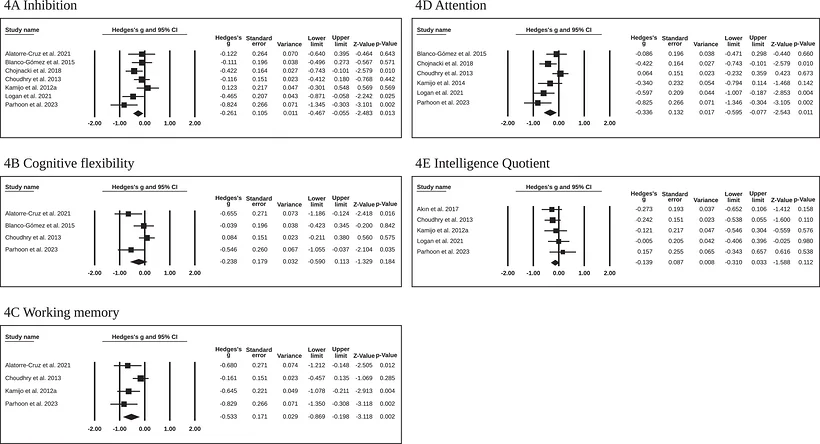
Forest plots displaying meta‐analytic effect sizes (Hedges’ *g* with 95% confidence intervals) for differences in cognitive performance between school‐aged children with obesity and their normal‐weight peers. Panels: (A) inhibition (*k* = 7); (B) cognitive flexibility (*k* = 4); (C) working memory (*k* = 4); (D) attention (*k* = 6); (E) IQ (*k* = 5). Negative effect sizes indicate poorer performance in the obesity group.

**FIGURE 5:**
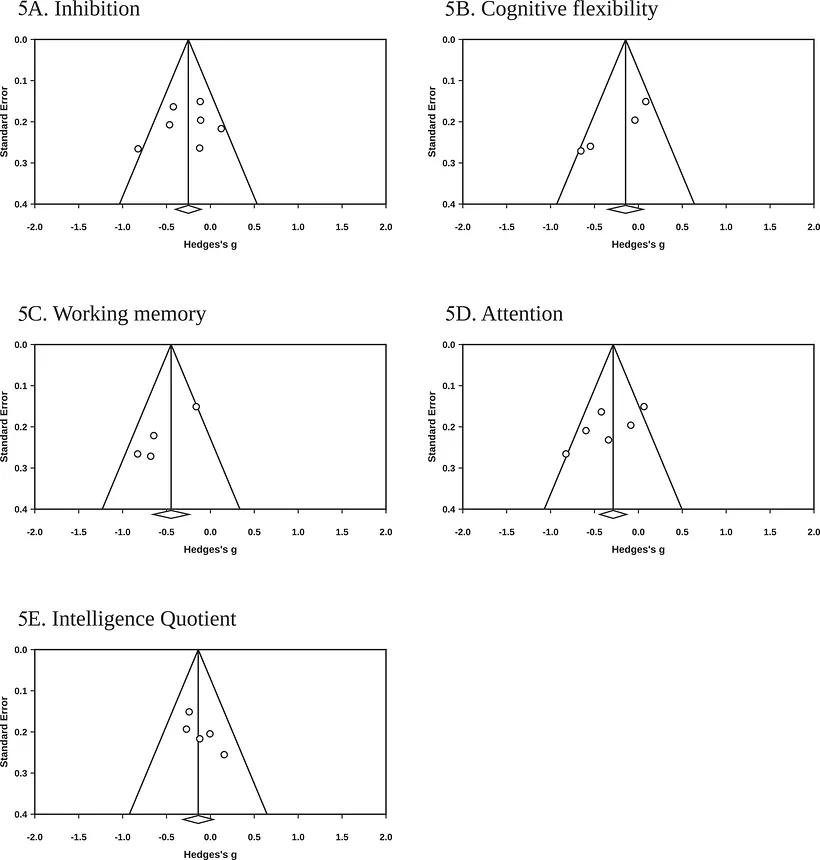
Funnel plots assessing publication bias for the school‐aged‐children subgroup across cognitive domains. Panels: (A) inhibition; (B) cognitive flexibility; (C) working memory; (D) attention; (E) IQ. Each plot displays individual study effect sizes (Hedges’ *g*) against their standard errors.

#### 3.4.2 Cognitive Flexibility

Across four studies, the effect on cognitive flexibility was non‐significant (*g* ^+^ = –0.238, *p* = 0.184, 95% CI [–0.590, 0.113]; Figure 4B), with substantial heterogeneity (*I* ^2^ = 64.9%). Egger's test indicated possible publication bias (*t*(2) = 6.197, *p* = 0.025), while Begg's test was non‐significant (*p* = 0.089; Figure 5B).

#### 3.4.3 Working Memory

Analysis of four studies revealed a significant effect (*g* ^+^ = –0.533, *p* = 0.002, 95% CI [–0.869, –0.198]; Figure 4C), indicating poorer working memory in school‐aged children with obesity. Heterogeneity was moderate (*I* ^2^ = 58.2%). Egger's test suggested possible publication bias (*t*(2) = 5.342, *p* = 0.033), though Begg's was non‐significant (*p* = 0.734; Figure 5C).

#### 3.4.4 Decision‐Making, Verbal Fluency, and Planning

Data were insufficient to examine group differences for these domains.

#### 3.4.5 Attention

Analysis of 6 studies showed a significant effect (*g* ^+^ = –0.336, *p* = 0.011, 95% CI [–0.595, –0.077]; Figure 4D), with school‐aged children with obesity performing worse on attention tasks. Moderate heterogeneity was observed (*I* ^2^ = 62.9%), and no publication bias was detected (Egger's *t*(4) = 1.955, *p* = 0.122; Begg's *p* = 0.133; Figure 5D).

#### 3.4.6 Intelligence Quotient (IQ)

Across five studies, the effect on IQ was non‐significant (*g* ^+^ = –0.139, *p* = 0.112, 95% CI [–0.310, 0.033]; Figure 4E), with no heterogeneity (*I* ^2^ = 0%). No publication bias was found (Egger's *t*(3) = 2.353, *p* = 0.100; Begg's *p* = 0.086; Figure 5E).

### 3.5 Subgroup Associations Between Obesity and Cognitive Function (Adolescents, 13–17 Years)

#### 3.5.1 Inhibition

Analysis of four studies showed a non‐significant overall effect of obesity on inhibition (*g* ^+^ = 0.006, *p* = 0.980, 95% CI [–0.497, 0.510]; Figure 6A), with high heterogeneity (*I* ^2^ = 77.1%). No publication bias was detected (Egger's *t*(2) = 0.366, *p* = 0.749; Begg's *p* = 0.734; Figure 7A).

**FIGURE 6:**
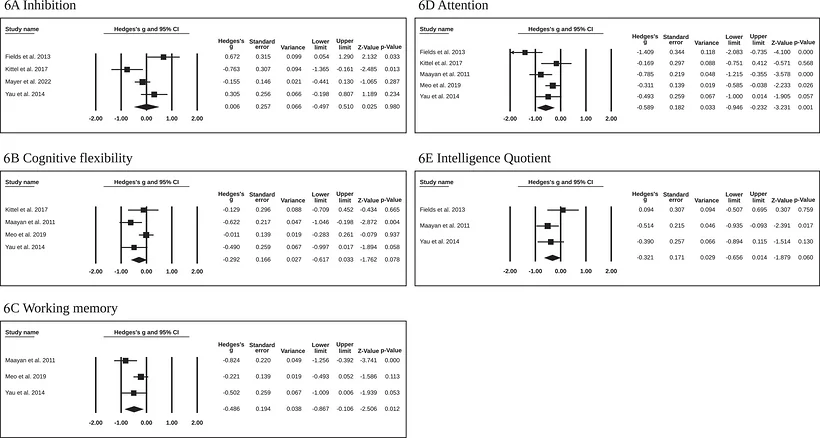
Forest plots displaying meta‐analytic effect sizes (Hedges’ *g* with 95% confidence intervals) for differences in cognitive performance between adolescents with obesity and normal‐weight adolescents. Panels: (A) inhibition (*k* = 4); (B) cognitive flexibility (*k* = 4); (C) working memory (*k* = 3); (D) attention (*k* = 5); (E) IQ (*k* = 3). Negative effect sizes indicate poorer performance in the obesity group.

**FIGURE 7:**
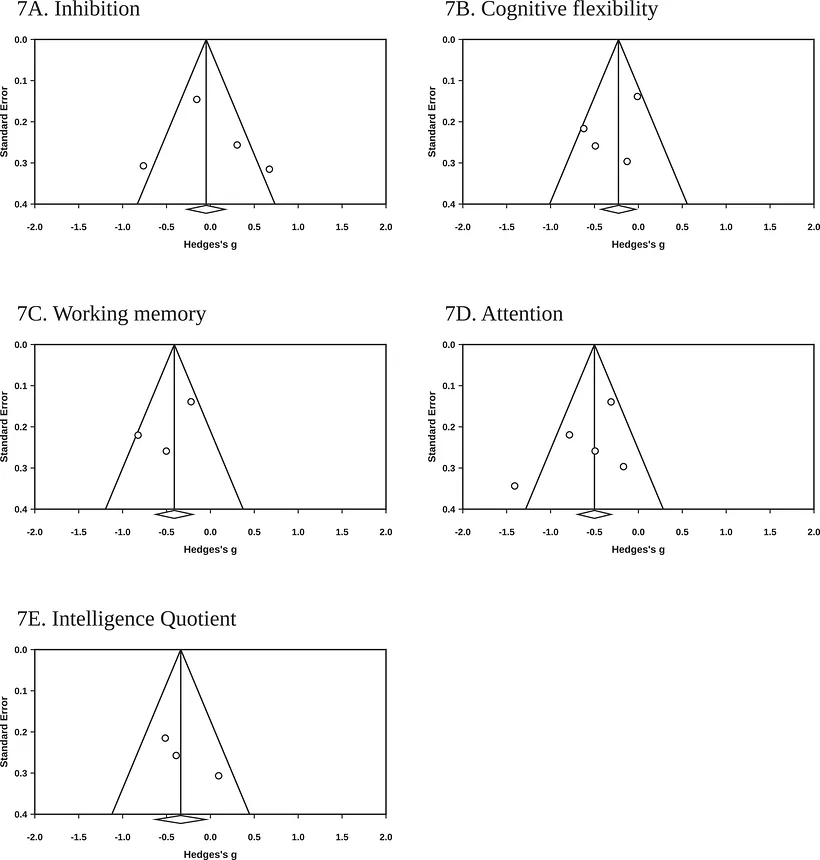
Funnel plots assessing publication bias for the adolescent subgroup analysis across cognitive domains. Panels: (A) inhibition; (B) cognitive flexibility; (C) working memory; (D) attention; (E) IQ. Each plot displays individual study effect sizes (Hedges’ *g*) against their standard errors.

#### 3.5.2 Cognitive Flexibility

Across four studies, the effect on cognitive flexibility was non‐significant (*g* ^+^ = –0.292, *p* = 0.078, 95% CI [–0.617, 0.033]; Figure 6B), with moderate heterogeneity (*I* ^2^ = 56.5%). No publication bias was found (Egger's *t*(2) = 1.093, *p* = 0.389; Begg's *p* = 1.000; Figure 7B).

#### 3.5.3 Working Memory

Analysis of three studies revealed a significant overall effect (*g* ^+^ = –0.486, *p* = 0.012, 95% CI [–0.867, –0.106]; Figure 6C), indicating poorer working memory in adolescents with obesity. Heterogeneity was moderate (*I* ^2^ = 63.7%). No publication bias was detected (Egger's *t*(1) = 1.278, *p* = 0.423; Begg's *p* = 1.000; Figure 7C).

#### 3.5.4 Decision‐Making, Verbal Fluency, and Planning

Data were insufficient to analyze differences in these domains.

#### 3.5.5 Attention

Analysis of five studies showed a significant overall effect of obesity on attention (*g* ^+^ = –0.589, *p* = 0.001, 95% CI [–0.946, –0.232]; Figure 6D), with moderate heterogeneity (*I* ^2^ = 66.0%). No publication bias was detected (Egger's *t*(3) = 1.228, *p* = 0.307; Begg's *p* = 0.462; Figure 7D).

#### 3.5.6 Intelligence Quotient (IQ)

Across three studies, the effect on IQ was non‐significant (*g* ^+^ = –0.321, *p* = 0.060, 95% CI [–0.656, 0.014]; Figure 6E), with low heterogeneity (*I* ^2^ = 25.8%). No publication bias was found (Egger's *t*(1) = 3.158, *p* = 0.195; Begg's *p* = 0.296; Figure 7E).

### 3.6 Publication Bias

Across the three sets of analyses (overall sample, school‐aged children, and adolescents), Egger's and Begg's tests were non‐significant for most cognitive domains, indicating little evidence of funnel‐plot asymmetry. Possible publication bias was detected for attention in the overall sample (Egger's *p* = 0.032; Begg's *p* = 0.038) and, based on Egger's test only, for cognitive flexibility (*p* = 0.025) and working memory (*p* = 0.033) in the school‐aged‐children subgroup; findings for these three domains should be interpreted with caution. Most domain‐ and subgroup‐specific analyses in this review are based on fewer than ten studies—the overall inhibition, cognitive flexibility, working memory, attention, and IQ analyses are the main exceptions, each including ten or more studies—and Egger's and Begg's tests are likely underpowered in these smaller domains, most notably verbal fluency (*k* = 3), planning (*k* = 3), working memory in adolescents (*k* = 3), IQ in adolescents (*k* = 3), and cognitive flexibility and working memory in the school‐aged‐children subgroup (*k* = 4 each). A non‐significant Egger's or Begg's test in these smaller domains should not be interpreted as ruling out publication bias (Sterne et al. 2011). As the attention domain was the only one with *k* ≥ 10 and significant bias tests, we further conducted a trim‐and‐fill sensitivity analysis (Duval and Tweedie 2000) for that domain. The *L*_0_ estimator imputed zero studies (*k*_0_ = 0), and the adjusted estimate (*g* ^+^ = −0.526, 95% CI [−0.738, −0.314]) was unchanged, suggesting the pooled attention effect is robust to publication bias.

### 3.7 Developmental‐Stage Comparison: Formal Test of Subgroup Differences

The subgroup analyses above show statistically significant obesity‐related deficits in some domains among school‐aged children but not among adolescents (most clearly for inhibition), which could be read as evidence that developmental stage moderates the obesity–cognition association. However, a difference in statistical significance between two subgroups is not itself evidence of a statistically significant difference between those subgroups. We therefore computed a formal test of subgroup differences for every domain with both a school‐aged‐children and an adolescent pooled estimate, using the mixed‐effects analog‐of‐ANOVA test for independent subgroups (the between‐groups *Q* statistic, *Q*‐between, 1 df; Borenstein et al. 2021): inhibition, *Q*(1) = 0.92, *p* = 0.34; cognitive flexibility, *Q*(1) = 0.05, *p* = 0.83; working memory, *Q*(1) = 0.03, *p* = 0.86; attention, *Q*(1) = 1.27, *p* = 0.26; IQ, *Q*(1) = 0.90, *p* = 0.34. None of these between‐subgroup differences reached statistical significance, including under a Bonferroni‐corrected threshold for the five domains tested (α = 0.05/5 = 0.01). School‐aged children and adolescents therefore did not differ reliably in the magnitude of the obesity–cognition association for any domain, despite the differing pattern of within‐subgroup significance reported above. The apparent developmental‐stage pattern in the subgroup analyses should accordingly be interpreted as descriptive rather than as evidence of moderation by developmental stage; this qualification is carried through to the Discussion.

## 4 Discussion

Executive functions are important for self‐regulation, learning, impulse control, and goal‐directed behavior. Although these abilities are highly relevant to obesity‐related behaviors, it remains unclear which cognitive domains are most consistently associated with obesity among youth. The present meta‐analysis addressed this issue by examining core executive function domains, including inhibition, cognitive flexibility, working memory, decision‐making, verbal fluency, and planning, together with broader neuropsychological outcomes such as attention and IQ.

Overall, the results suggest that obesity is associated with poorer cognitive performance across several domains (Table 5). Across all included studies, youth with obesity showed lower performance than normal‐weight controls in attention, IQ, inhibition, cognitive flexibility, working memory, and verbal fluency. Subgroup analyses showed that school‐aged children with obesity had significant deficits mainly in inhibition, working memory, and attention, while in adolescents significant differences were observed mainly in working memory and attention; however, as detailed below, formal between‐subgroup tests found no statistically significant difference between school‐aged children and adolescents for any domain, so this apparent developmental contrast should not yet be read as established moderation.

**TABLE 5:** Summary of meta‐analytic effect sizes (hedge's g) for cognitive domains.

Group	ALL				School‐aged children (6–12 years)				Adolescents (13–17 years)			
Variable(s)	Hedge's *g*	Lower limit	Upper limit	*P*‐value	Hedge's *g*	Lower limit	Upper limit	*P*‐value	Hedge's *g*	Lower limit	Upper limit	*P*‐value
Inhibition	**−0.193**	**−0.386**	**−0.001**	**0.049**	**−0.261**	**−0.467**	**−0.055**	**0.013**	0.006	−0.497	0.510	0.980
Cognitive flexibility	−**0.499**	−**0.849**	−**0.149**	**0.005**	−0.238	−0.590	0.113	0.184	−0.292	−0.617	0.033	0.078
Working memory	−**0.652**	−**0.866**	−**0.438**	**<0.0001**	−**0.533**	−**0.869**	−**0.198**	**0.002**	−**0.486**	−**0.867**	−**0.106**	**0.012**
Decision‐making	N/A				N/A				N/A			
Verbal fluency	−**0.420**	−**0.695**	−**0.145**	**0.003**	N/A				N/A			
Planning	−0.638	−1.527	0.250	0.159	N/A				N/A			
Attention	−**0.526**	−**0.738**	−**0.314**	**<0.0001**	−0.336	−0.595	−0.077	**0.011**	−**0.589**	−**0.946**	−**0.232**	**0.001**
Intelligence quotient	−**0.288**	−**0.429**	−**0.148**	**<0.0001**	−0.139	−0.310	0.033	0.112	−0.321	−0.656	0.014	0.060

*Note*: This table serves as the primary outcome of the meta‐analysis, comparing performance between NW and OB groups across all EF domains. It reports Hedge's *g* (effect size), 95% confidence intervals (lower and upper limits), and *p*‐values.

Across all age groups, however, the limited number of available studies made it difficult to draw definitive conclusions regarding weight‐related differences in decision‐making, verbal fluency, and planning.

### 4.1 Discussion of Moderators

We further examined factors that might influence the association between obesity and cognitive functioning, testing mean age and percentage of female participants as moderators for the four domains with sufficient studies (inhibition, working memory, attention, and IQ; Table 4), for a total of seven moderator tests (mean age was not tested for inhibition; Table 4). Among these, the percentage of female participants was a statistically significant moderator only for working memory (*p* = 0.002; Table 4); this result survives a conservative Bonferroni correction for multiple comparisons (adjusted α = 0.05/7 ≈ 0.007), but given the exploratory nature of the broader set of moderator analyses conducted in this meta‐analysis, it should still be treated as provisional pending replication.

This finding indicates a study‐level (ecological) association between the proportion of female participants in a sample and the magnitude of the obesity–working‐memory effect; it does not establish that the association operates at the level of individual girls versus boys, and inferring an individual‐level, sex‐specific mechanism from a study‐level correlation risks an ecological‐inference fallacy. With that caveat made explicit, one plausible explanation is that it reflects sex‐related differences in brain maturation and hormonal regulation during development: prior research indicates that girls tend to show earlier maturation of the prefrontal cortex and related executive networks, including systems involved in working memory, compared with boys during childhood and adolescence (Gur and Gur 2016; Lenroot and Giedd 2010). However, alternative, non‐biological explanations are equally plausible and cannot be ruled out with the available data: studies that recruited a higher proportion of female participants may have differed systematically in the working‐memory tasks used, the clinical or socioeconomic characteristics of their samples, or their recruitment sources, any of which could produce the observed pattern independent of individual‐level sex differences. This moderation effect was not found for inhibition, attention, or IQ (Table 4), indicating that any such influence, biological or methodological, is domain‐specific rather than uniform across neurocognitive outcomes. Future primary studies should report sex‐stratified, individual‐level analyses to help distinguish between these explanations.

We also directly tested whether developmental stage itself moderated the obesity–cognition association, beyond the descriptive contrast between subgroup significance patterns reported above. A formal between‐subgroup test (see Section 3, Developmental‐stage comparison) found no statistically significant difference between school‐aged children and adolescents for any domain (all *p* ≥ 0.26, including for inhibition, where the raw subgroup estimates differed most visibly). This indicates that developmental stage, unlike the proportion of female participants, is not demonstrated to be a reliable moderator of the obesity–cognition association in these data; the pattern of significance in some domains among school‐aged children but not adolescents more likely reflects differences in statistical power between subgroups of unequal size (*k* = 4–7 vs. *k* = 3–6) than a true difference in effect magnitude. We treat this developmental pattern as descriptive rather than confirmatory throughout the remainder of the Discussion.

### 4.2 Biological and Structural Mechanisms

Biological and neurodevelopmental mechanisms may help explain the association between obesity and executive functioning. Structural neuroimaging evidence is broadly consistent with a brain‐based pathway: in a large sample of 9‐ to 11‐year‐olds, higher BMI was associated with reduced cortical thickness in prefrontal regions, which partially mediated the association between BMI and executive function performance (Ronan et al. 2020). Using a similarly large pediatric cohort, Li et al. (2023) found that childhood obesity and lower cognitive performance were both associated with, and appeared to partially mediate, socioeconomic disparities in white‐matter microstructure, suggesting that obesity‐related brain differences may not be independent of broader social and environmental exposures. At the level of specific cognitive mechanisms, Herzog et al. (2024) found, in a sample of adults, that higher BMI was associated with poorer working‐memory gating and that this association was moderated by striatal dopaminergic gene variants, implicating reward‐related neurotransmitter systems as a further candidate pathway distinct from prefrontal structure alone. Lane et al. (2023) additionally proposed the gut–microbiome–brain axis as a pathway through which adolescent obesity and executive function may influence each other, though this remains more speculative than the structural and genetic evidence above. Earlier meta‐analytic work in pediatric samples (Pearce, Leonhardt, et al. 2018) similarly reported executive and reward‐related differences associated with obesity, reinforcing that the pattern observed here is consistent with the broader literature rather than an isolated finding. Taken together, these findings suggest that obesity‐related cognitive differences in youth likely reflect a combination of structural, neurochemical, and social‐environmental processes rather than a single mechanism, and that these processes may interact bidirectionally with obesity itself rather than obesity acting as a simple, unidirectional cause. Future studies in youth that combine metabolic, microbiome, structural neuroimaging, and neurocognitive measures, ideally with longitudinal or genetically informed designs, would help clarify these mechanisms.

### 4.3 Comparison With a Concurrent Meta‐Analysis

While this review was in preparation, Ásgeirsdóttir et al. (2026) published a preregistered meta‐analysis of executive function in adolescents with obesity that overlaps substantially with the present review in domains, moderators, and included studies. Several points of comparison are relevant. On overall magnitude, Ásgeirsdóttir et al. (2026) reported a small, domain‐general executive‐function deficit (*g* = −0.314) that did not differ significantly across specific domains, whereas the present review found domain‐specific overall effects ranging from non‐significant (decision‐making, not estimable) to moderate (working memory, *g* = −0.652); this difference likely reflects the present review's inclusion of school‐aged children alongside adolescents and its extension to attention and IQ, neither of which was examined by Ásgeirsdóttir et al. (2026). For working memory specifically, our adolescent‐only subgroup estimate (*g* = −0.486, *k* = 3) is directionally consistent with, though less precisely estimated than, the broader effect reported by Ásgeirsdóttir et al. (2026), supporting working memory as one of the more robust domains in this literature. For inhibition, by contrast, our adolescent‐only subgroup estimate was null (*g* = 0.006, *p* = 0.980, *k* = 4), diverging from the general deficit Ásgeirsdóttir et al. (2026) reported; given the small number of adolescent‐only inhibition studies in our sample, this discrepancy plausibly reflects sampling variability rather than a genuine difference in findings and should not be read as evidence against an inhibition deficit in adolescents with obesity. On the moderating role of age, Ásgeirsdóttir et al. (2026) reported that samples with higher age showed more pronounced weight‐group differences, whereas our formal test of developmental‐stage differences (see Section 3 and above) found no statistically significant difference between school‐aged children and adolescents for any domain. These findings are not necessarily contradictory: Ásgeirsdóttir et al. (2026) modeled age continuously within an adolescent‐only sample, whereas the present review compared two discrete developmental groups spanning a wider age range, and continuous and categorical moderator analyses can diverge even within overlapping literatures, particularly at the modest per‐domain sample sizes common to both reviews. The present review's distinct contribution is therefore not to supersede Ásgeirsdóttir et al. (2026) but to extend the evidence base to school‐aged children and to two additional outcome domains, attention and IQ, that adolescent‐only, executive‐function‐focused reviews do not address.

### 4.4 Methodological Limitations Related to BMI Classification and Grouping

A major methodological issue in this meta‐analysis is the variation in BMI classification criteria across the included studies, which may have affected the validity and interpretation of the main findings. While most studies used the ≥ 95th percentile as the threshold for obesity, some adopted stricter criteria (e.g., ≥ 97th percentile) or applied absolute BMI cutoffs (e.g., BMI > 30 kg/m^2^), while others used different reference systems such as WHO, CDC, or IOTF standards. Obesity definitions based on BMI *Z*‐scores differ by reference system. The IOTF defines obesity in children aged 2–18 as *Z*‐scores above +2.29 for boys and +2.19 for girls. The WHO defines obesity in those aged 5–19 as *Z*‐scores above +2 for both sexes. The CDC defines obesity in children aged 2–20 as *Z*‐scores above +1.64, regardless of sex (Cole and Lobstein 2023).

Variations in obesity criteria may have resulted in differences in the severity and clinical characteristics of participants classified as having obesity across studies. These differences likely contributed to heterogeneity in the pooled estimates and made comparisons of effect sizes less straightforward. A related limitation is that more than 20 studies grouped participants with overweight and obesity together as a single OV+OB category. Because separate data were not available for each subgroup, these studies could not be included in either the overweight‐specific or obesity‐specific analyses. This reduced the number of studies available for direct comparisons and may have limited the precision of the estimates. It may also have masked meaningful differences between groups, as individuals with overweight generally show milder cognitive and metabolic differences than individuals with obesity.

Therefore, pooling these two weight categories together might weaken the observed deficits in the obesity group or hide subtle cognitive changes occurring in the overweight group. These differences in BMI definitions and the frequent merging of weight categories limit the precision of current meta‐analytic estimates. Future primary research should use consistent classification standards and report separate results for overweight and obesity to allow for more accurate and developmentally sensitive comparisons.

### 4.5 Developmental Differences and Age‐Specific Patterns in Cognitive Outcomes

This meta‐analysis compared an overall analysis combining all participants under 18 years with subgroup analyses contrasting school‐aged children and adolescents (Table 5). As reported above, within‐subgroup significance differed somewhat between the two developmental groups, but formal between‐subgroup tests found no statistically significant difference for any domain; the apparent age‐related pattern is therefore best read as descriptive.

Some primary studies included both children and adolescents but did not report separate results for each age group. To maintain statistical independence and avoid double‐counting, these mixed‐age studies were included only in the overall analyses and excluded from the age‐specific subgroup analyses. This is also why, for several domains, the overall *k* exceeds the sum of the two subgroup *k*’s (see Section 3.1). Although this approach was necessary to avoid double‐counting, it means that some age‐related differences may not have been fully captured. Mixed‐age samples may produce average estimates that do not clearly reflect differences between children and adolescents. Weaker effects in one age group could reduce the estimated effect in another, while stronger effects in younger participants could make the differences in older groups appear larger.

Removing these mixed‐age studies from the subgroup analyses also reduced statistical power in those smaller subgroups, which, combined with the non‐significant formal subgroup‐difference tests reported above, means that any true developmental‐stage differences in the obesity–cognition association remain inconclusively tested with the current evidence base rather than demonstrated. Future primary studies should publish age‐stratified data to support more precise developmental modeling in subsequent meta‐analyses.

### 4.6 Strengths and Clinical Contributions

Our meta‐analysis advances the current research by addressing several key gaps in previous reviews. First, our much larger sample size of individuals with obesity significantly boosts statistical power. Second, earlier meta‐analyses often limited their scope to a single executive function (Wu et al. 2016), one cognitive task (Lavagnino et al. 2016), or ignored broader neuropsychological measures (Yang et al. 2018). In contrast, we evaluated a wider range of cognitive and neuropsychological markers to paint a more complete cognitive picture. Third, we used rigorous subgroup analyses across different developmental stages to map differences against normal‐weight controls. Finally, we examined demographic moderators and identified the sex composition of study samples as a study‐level correlate of the obesity–working‐memory association, while treating candidate biological mechanisms and causal direction as questions for future, ideally longitudinal, research rather than as conclusions of the present design.

### 4.7 Limitations and Future Directions

First, the limited number of studies examining the association between obesity and verbal fluency or planning hindered our ability to make inferences about the effects of obesity on these executive functions, particularly concerning potential moderators. Similarly, too few studies exist to properly compare how individuals with obesity and normal‐weight individuals perform on decision‐making tasks. Because of these gaps, once more data becomes available on verbal fluency, planning, or decision‐making in relation to obesity, a follow‐up meta‐analysis will be highly justified.

Second, there may be moderators of the associations between obesity and cognitive functions that are unaccounted for in our analyses. Some studies failed to report important information, such as BMI percentile, education, economic status, the clinical status of participants, and whether participants took any obesity‐treating medication, which precluded moderator analyses on these variables. As such, we do not claim to provide a comprehensive account of the moderators influencing the associations between obesity and executive or neuropsychological functions. Future large‐scale studies should aim to thoroughly characterize participants and examine whether additional factors moderate the association between excess weight and executive and neuropsychological functioning.

Third, another limitation of this meta‐analysis is the inconsistency in BMI classification and grouping across the included studies. Over 20 studies did not differentiate between overweight and obesity, instead combining them into a single group (OV+OB). As these studies did not report separate data for each subgroup, they could not be included in overweight‐ or obesity‐specific analyses, which reduced the sample size and statistical power for subgroup comparisons. Moreover, the heterogeneity in BMI definitions and the frequent merging of weight categories may have compromised the precision and interpretability of the findings regarding the association between weight status and cognitive outcomes.

Fourth, several included studies lacked age‐stratified data, which prevented separate analyses for children and adolescents. As a result, mixed‐age samples were only included in overall analyses, potentially obscuring developmental differences. Since executive function and attention mature at different rates, combining age groups may have underestimated or diluted age‐specific cognitive effects. Moreover, only one included study sampled preschool children, so the younger subgroup is confined to school‐aged children (6–12 years) and the early‐childhood period remains essentially unexamined. Future studies should report stratified results by developmental stage, including preschool samples, to enable more accurate comparisons.

Fifth, although obesity and ADHD are theoretically linked through overlapping executive‐function pathways (see Introduction), only two included studies directly compared youth with obesity and comorbid ADHD to relevant controls, and their findings diverged (see Section 3.2). This precluded a quantitative moderator analysis of ADHD status, which had originally been considered as a possible moderator for this review, and represents an important gap for future primary research that combines obesity and ADHD phenotyping within the same samples.

Sixth, this systematic review and meta‐analysis was not prospectively registered (e.g., in PROSPERO). Although the review followed PRISMA reporting guidelines throughout, adherence to a reporting guideline does not substitute for prospective registration, which helps guard against selective outcome reporting and undisclosed post hoc changes to the review protocol. This should be considered when interpreting the findings and is a target for improvement in future updates of this review.

## 5 Conclusion

This meta‐analysis found that youth obesity is associated with poorer performance across several cognitive domains, including inhibition, cognitive flexibility, working memory, verbal fluency, attention, and IQ—although the inhibition effect was small and only marginally significant (*g* ^+^ = –0.193, *p* = 0.049, 95% CI [–0.386, –0.001]), and several of these estimates were based on as few as three studies. While associations were observed in both children and adolescents, formal between‐subgroup tests found no statistically significant difference between developmental stages for any domain, so the present findings offer preliminary, descriptive evidence about how the obesity–cognition association might vary across development rather than confirmed developmental moderation.

By focusing on individuals under 18 years old, this study extends earlier meta‐analyses to provide a more detailed picture of the cognitive correlates of obesity in young people. Our moderator analysis also indicated that the sex composition of study samples was associated with the magnitude of the obesity–working‐memory association, highlighting sex composition as a factor worth accounting for in future research—although, as noted above, this study‐level association should not be over‐interpreted as an individual‐level, sex‐specific effect.

These findings should be interpreted with caution given several methodological limitations: substantial statistical heterogeneity in several domains; predominantly cross‐sectional and observational study designs that cannot establish the direction of any association; evidence of possible publication bias for attention and for two domains in the younger subgroup; small numbers of studies in some domains (verbal fluency, planning, adolescent working memory, and adolescent IQ); heterogeneity in BMI classification; limited age‐ and ADHD‐stratified data; multiple moderator comparisons conducted across domains; and reliance on BMI as a sole measure of adiposity. Because the included studies were almost entirely cross‐sectional, the associations reported here should be understood as bidirectional or as‐yet‐undetermined in direction rather than as evidence that obesity causes cognitive impairment; it remains equally plausible that early cognitive or self‐regulatory differences contribute to the development of obesity, or that both are influenced by shared biological, environmental, or socioeconomic factors.

Future studies should use more consistent weight classification criteria, report separate data by developmental stage, sex, and comorbid conditions such as ADHD, and adopt longitudinal or genetically informed designs capable of testing the direction of these associations. This would make findings easier to compare across studies and would help clarify how obesity and cognitive functioning in youth relate to one another, ultimately supporting more targeted prevention and intervention strategies.

## Author Contributions

**Yi‐Chun Lin**: writing – original draft, conceptualization, methodology, formal analysis. **Chia‐Chia Chang**: conceptualization, investigation, writing – original draft, methodology, formal analysis, data curation. **Yu‐Tzu Chang**: conceptualization, methodology, formal analysis, supervision, resources, writing – review and editing. **Wei‐Lin Liu**: writing – review and editing, validation, data curation, software, resources. **Yu‐Chi Liao**: conceptualization, investigation, funding acquisition, writing – original draft, writing – review and editing, validation, software, project administration, resources, supervision, visualization, formal analysis. **Ro‐Ting Lin**: writing – review and editing, funding acquisition, investigation, conceptualization, methodology, validation, software, formal analysis, project administration, supervision, resources.

## Funding

This study was supported by Asia University and China Medical University Hospital (grant numbers: ASIA‐112‐CMUH‐03 and ASIA‐111‐CMUH‐12), National Science and Technology Council, Taiwan (grant numbers: NSTC 114‐2410‐H‐468‐023‐MY3).

## Ethics Statement

Ethical approval and informed consent were not required for this study because it was a systematic review and meta‐analysis based exclusively on data from previously published studies and involved no direct participation of human subjects or collection of identifiable personal information.

## Impact of the Study

• The study examines individuals under 18 across developmental stages with subgroup comparisons, highlighting age‐specific patterns in the cognitive correlates of obesity.

• By integrating the evaluation of multiple cognitive domains (inhibition, cognitive flexibility, working memory, decision‐making, verbal fluency, IQ, and attention), the study provides a comprehensive picture of cognitive performance differences associated with obesity in youth.

• By restricting inclusion to studies reporting separate obesity groups, this study provides a more precise estimate of cognitive differences associated with obesity specifically, rather than combining overweight and obese samples.

## Conflicts of Interest

The authors declare no conflicts of Interest.

## Supporting information




**Supplementary Materials**: brb371884‐sup‐0001‐SuppMat.docx

## Data Availability

To support open data practices for meta‐analytic research, the full coded dataset will also be deposited in a repository. (https://github.com/Kelvinycliao/CogFuncYouthObesity)
