# Supplementary material for: The Cognitive Functions of Youth With Obesity: A Meta‐Analysis
Source: Brain Behav. 2026 Nov 1;16(11):e71884. doi: 10.1002/brb3.71884 (PMC13724984; doi:10.1002/brb3.71884)
Supplement: Supplementary file 1 [file BRB3-16-e71884-s001.docx]

# Table S1

*PRISMA 2020 checklist*

| **Section and Topic** | **Item #** | **Checklist item** | **Location where item is**  **reported** |
| --- | --- | --- | --- |
| **TITLE** | | |  |
| Title | 1 | Identify the report as a systematic review. | N/A |
| **ABSTRACT** | | |  |
| Abstract | 2 | See the PRISMA 2020 for Abstracts checklist. | p.3 |
| **INTRODUCTION** | | |  |
| Rationale | 3 | Describe the rationale for the review in the context of existing knowledge. | p.2 |
| Objectives | 4 | Provide an explicit statement of the objective(s) or question(s) the review addresses. | p.4-7 |
| **METHODS** | | |  |
| Eligibility criteria | 5 | Specify the inclusion and exclusion criteria for the review and how studies were grouped for the syntheses. | p.8-9  Table 1 |
| Information sources | 6 | Specify all databases, registers, websites, organisations, reference lists and other sources searched or consulted to identify studies. Specify the date when each source was last searched or consulted. | p.8 |
| Search strategy | 7 | Present the full search strategies for all databases, registers and websites, including any filters and limits used. | Table S2 |

| **Section and Topic** | **Item #** | **Checklist item** | **Location where item is**  **reported** |
| --- | --- | --- | --- |
| Selection process | 8 | Specify the methods used to decide whether a study met the inclusion criteria of the review, including how many reviewers screened each record and each report retrieved, whether they worked independently, and if applicable,  details of automation tools used in the process. | Figure 1 |
| Data  collection process | 9 | Specify the methods used to collect data from reports, including how many reviewers collected data from each report,  whether they worked independently, any processes for obtaining or confirming data from study investigators, and if applicable, details of automation tools used in the process. | Figure 1 |
| Data items | 10a | List and define all outcomes for which data were sought. Specify whether all results that were compatible with each  outcome domain in each study were sought (e.g. for all measures, time points, analyses), and if not, the methods used to decide which results to collect. | p.8-10 |
|  | 10b | List and define all other variables for which data were sought (e.g. participant and intervention characteristics, funding sources). Describe any assumptions made about any missing or unclear information. | p.8-10 |
| Study risk of bias assessment | 11 | Specify the methods used to assess risk of bias in the included studies, including details of the tool(s) used, how many reviewers assessed each study and whether they worked independently, and if applicable, details of automation tools used in the process. | p.9-10 |
| Effect measures | 12 | Specify for each outcome the effect measure(s) (e.g. risk ratio, mean difference) used in the synthesis or presentation of results. | p.9-10 |
| Synthesis | 13a | Describe the processes used to decide which studies were eligible for each synthesis (e.g. tabulating the study | p.9-10 |

| **Section and Topic** | **Item #** | **Checklist item** | **Location where item is**  **reported** |
| --- | --- | --- | --- |
| methods |  | intervention characteristics and comparing against the planned groups for each synthesis (item #5)). |  |
|  | 13b | Describe any methods required to prepare the data for presentation or synthesis, such as handling of missing summary statistics, or data conversions. | p.9-10 |
|  | 13c | Describe any methods used to tabulate or visually display results of individual studies and syntheses. | p.9-10 |
|  | 13d | Describe any methods used to synthesize results and provide a rationale for the choice(s). If meta-analysis was  performed, describe the model(s), method(s) to identify the presence and extent of statistical heterogeneity, and software package(s) used. | p.10 |
|  | 13e | Describe any methods used to explore possible causes of heterogeneity among study results (e.g. subgroup analysis, meta-regression). | p.9-10 |
|  | 13f | Describe any sensitivity analyses conducted to assess robustness of the synthesized results. | Results: Attention subsection (Publication Bias; Duval & Tweedie, 2000 trim-and-fill) |
| Reporting bias assessment | 14 | Describe any methods used to assess risk of bias due to missing results in a synthesis (arising from reporting biases). | p.10 |
| Certainty assessment | 15 | Describe any methods used to assess certainty (or confidence) in the body of evidence for an outcome. | p.9-10 |
| **RESULTS** | | |  |

| **Section and Topic** | **Item #** | **Checklist item** | **Location where item is**  **reported** |
| --- | --- | --- | --- |
| Study selection | 16a | Describe the results of the search and selection process, from the number of records identified in the search to the  number of studies included in the review, ideally using a flow diagram. | Figure 1 |
|  | 16b | Cite studies that might appear to meet the inclusion criteria, but which were excluded, and explain why they were excluded. | Figure 1 |
| Study characteristics | 17 | Cite each included study and present its characteristics. | p.11  Table 2 |
| Risk of bias in studies | 18 | Present assessments of risk of bias for each included study. | Table S3 |
| Results of individual studies | 19 | For all outcomes, present, for each study: (a) summary statistics for each group (where appropriate) and (b) an effect estimate and its precision (e.g. confidence/credible interval), ideally using structured tables or plots. | Table 5  p.10 |
| Results of syntheses | 20a | For each synthesis, briefly summarise the characteristics and risk of bias among contributing studies. | p.11-15 |
|  | 20b | Present results of all statistical syntheses conducted. If meta-analysis was done, present for each the summary estimate  and its precision (e.g. confidence/credible interval) and measures of statistical heterogeneity. If comparing groups, describe the direction of the effect. | p.11-15 |
|  | 20c | Present results of all investigations of possible causes of heterogeneity among study results. | p.11-15 |

| **Section and Topic** | **Item #** | **Checklist item** | **Location where item is**  **reported** |
| --- | --- | --- | --- |
|  | 20d | Present results of all sensitivity analyses conducted to assess the robustness of the synthesized results. | Results: Attention subsection and Publication Bias subsection (trim-and-fill, k₀ = 0) |
| Reporting biases | 21 | Present assessments of risk of bias due to missing results (arising from reporting biases) for each synthesis assessed. | p.11-15 |
| Certainty of evidence | 22 | Present assessments of certainty (or confidence) in the body of evidence for each outcome assessed. | p.11-15 |
| **DISCUSSION** | | |  |
| Discussion | 23a | Provide a general interpretation of the results in the context of other evidence. | p.16-21 |
|  | 23b | Discuss any limitations of the evidence included in the review. | p.20-21 |
|  | 23c | Discuss any limitations of the review processes used. | p.20-21 |
|  | 23d | Discuss implications of the results for practice, policy, and future research. | p.20-21 |
| **OTHER INFORMATION** | | |  |
| Registration and protocol | 24a | Provide registration information for the review, including register name and registration number, or state that the review was not registered. | The review was not registered; this is stated explicitly in the Methods and acknowledged as Limitation 6 in the Discussion. |
|  | 24b | Indicate where the review protocol can be accessed, or state that a protocol was not prepared. | No review protocol was prepared or separately published. |
|  | 24c | Describe and explain any amendments to information provided at registration or in the protocol. | N/A |

| **Section and Topic** | **Item #** | **Checklist item** | **Location where item is**  **reported** |
| --- | --- | --- | --- |
| Support | 25 | Describe sources of financial or non-financial support for the review, and the role of the funders or sponsors in the  review. | Funding statement (manuscript p.1) |
| Competing interests | 26 | Declare any competing interests of review authors. | Competing interests statement (manuscript p.22) |
| Availability of  data, code and other materials | 27 | Report which of the following are publicly available and where they can be found: template data collection forms; data extracted from included studies; data used for all analyses; analytic code; any other materials used in the review. | Data Availability Statement (manuscript p.1); study-level data in Tables 2–5 and Supplementary Materials; coded dataset: https://github.com/Kelvinycliao/CogFuncYouthObesity |

*From:* Page MJ, McKenzie JE, Bossuyt PM, Boutron I, Hoffmann TC, Mulrow CD, et al. The PRISMA 2020 statement: an updated guideline for reporting systematic reviews. BMJ 2021;372:n71. doi: 10.1136/bmj.n71. This work is licensed under CC BY 4.0. To view a copy of this license, visit https://creativecommons.org/licenses/by/4.0/

# Table S2

*Search strategy in each database*

Database	Search Strategy	Search Results

Medline	(1) TX "infant" OR TX "preschool child" OR TX "child" OR TX "adolescent" OR TX "child, preschool" OR TX "pre-school child" OR TX "pre-school going children" OR TX "pre-schooler" OR TX "pre-schoolers" OR TX "preschool child institution" OR TX "preschooler" OR TX "children" OR TX "teenager"

(2) TX "obesity" OR TX "childhood obesity" OR TX "adolescent obesity" OR TX "adipose tissue hyperplasia" OR TX "adipositas" OR TX "adiposity" OR TX "alimentary obesity" OR TX "body weight, excess" OR TX "corpulency" OR TX "fat overload syndrome" OR TX "nutritional obesity" OR TX "obesitas" OR TX "overweight" OR TX "paediatric obesity" OR TX "pediatric obesity"
(3) TX "attention test" OR TX "intelligence test" OR TX "executive function test" OR TX "neuropsychological assessment" OR TX "intelligence measurement" OR TX "intelligence tests" OR TX "measurement, intelligence" OR TX "executive function task" OR TX "problem solving task" OR TX "problem solving test" OR TX "neuro-psychological assessment" OR TX "neuro-psychological examination" OR TX "neuro- psychological test" OR TX "neuropsychologic test" OR TX "neuropsychological examination" OR TX "neuropsychological test" OR TX "neuropsychological tests" OR TX "neuropsychology test" OR TX "test, neuropsychological"
(4) (1) AND (2) AND (3)

Embase	(1) "infant"/exp OR "preschool child"/exp OR "child"/exp OR "adolescent"/exp OR "child, preschool"/ti,ab,kw OR	"pre-school child"/ti,ab,kw OR "pre-school going children"/ti,ab,kw OR "pre-schooler"/ti,ab,kw OR "pre- schoolers"/ti,ab,kw OR "preschool child institution"/ti,ab,kw OR "preschooler"/ti,ab,kw" OR

7837

657

"children"/ti,ab,kw OR "teenager"/ti,ab,kw

(2) "obesity"/exp OR "childhood obesity"/exp OR "adolescent obesity"/exp OR "adipose tissue hyperplasia"/ti,ab,kw OR "adipositas"/ti,ab,kw OR "adiposity"/ti,ab,kw OR "alimentary obesity"/ti,ab,kw OR "body weight, excess"/ti,ab,kw OR "corpulency"/ti,ab,kw OR "fat overload syndrome"/ti,ab,kw OR "nutritional obesity"/ti,ab,kw OR "obesitas"/ti,ab,kw OR "overweight"/ti,ab,kw OR "paediatric obesity"/ti,ab,kw OR "pediatric obesity"/ti,ab,kw
(3) "attention test"/exp OR "intelligence test"/exp OR "executive function test"/exp OR "neuropsychological assessment"/exp OR "intelligence measurement"/ti,ab,kw OR "intelligence tests"/ti,ab,kw OR "measurement, intelligence"/ti,ab,kw OR "executive function task"/ti,ab,kw OR "problem solving task"/ti,ab,kw OR "problem solving test"/ti,ab,kw OR "neuro-psychological assessment"/ti,ab,kw OR "neuro-psychological examination"/ti,ab,kw OR "neuro-psychological test"/ti,ab,kw OR "neuropsychologic test"/ti,ab,kw OR "neuropsychological examination"/ti,ab,kw OR "neuropsychological test"/ti,ab,kw OR "neuropsychological tests"/ti,ab,kw OR "neuropsychology test"/ti,ab,kw OR "test, neuropsychological"/ti,ab,kw
(4) (1) AND (2) AND (3)

PubMed	(1) "infant"[MeSH Terms] OR "child, preschool"[MeSH Terms] OR "Child"[MeSH Terms] OR "Adolescent"[MeSH Terms] OR "Infants"[Text Word] OR "preschool child"[Text Word] OR "children preschool"[Text Word] OR "preschool children"[Text Word] OR "Children"[Text Word] OR "Adolescents"[Text Word] OR "Adolescence"[Text Word] OR "Teens"[Text Word] OR "Teen"[Text Word] OR "Teenagers"[Text Word] OR "Teenager"[Text Word] OR "Youth"[Text Word] OR "Youths"[Text

1065

Word] OR "adolescents female"[Text Word] OR "adolescent female"[Text Word] OR "female adolescent"[Text Word] OR "female adolescents"[Text Word] OR "adolescents male"[Text Word] OR "adolescent male"[Text Word] OR "male adolescent"[Text Word] OR "male adolescents"[Text Word]

(2) "obesity"[MeSH Terms] OR "pediatric obesity"[MeSH Terms] OR "Overweight"[MeSH Terms] OR "obesity pediatric"[Text Word] OR "obesity in childhood"[Text Word] OR "childhood onset obesity"[Text Word] OR "obesity childhood onset"[Text Word] OR "child obesity"[Text Word] OR "obesity child"[Text Word] OR "childhood obesity"[Text Word] OR "obesity childhood"[Text Word] OR "adolescent obesity"[Text Word] OR "obesity adolescent"[Text Word] OR "obesity in adolescence"[Text Word] OR "childhood overweight"[Text Word] OR "overweight childhood"[Text Word] OR "adolescent overweight"[Text Word] OR "overweight adolescent"[Text Word]
(3) "attention"[MeSH Terms] OR "intelligence tests"[MeSH Terms] OR "executive function"[MeSH Terms] OR "neuropsychological tests"[MeSH Terms] OR "focus of attention"[Text Word] OR "attention focus"[Text Word] OR "social attention"[Text Word] OR "attention social"[Text Word] OR (("Attention"[MeSH Terms] OR "Attention"[All Fields] OR "attentions"[All Fields] OR "attention s"[All Fields] OR "attentional"[All Fields] OR "attentive"[All Fields] OR "attentively"[All Fields] OR "attentiveness"[All Fields]) AND "Social"[Text Word]) OR "intelligence test"[Text Word] OR "test intelligence"[Text Word] OR "raven s progressive matrices"[Text Word] OR "raven progressive matrices"[Text Word] OR "ravens progressive matrices"[Text Word] OR "raven test"[Text Word] OR "test raven"[Text Word] OR "mental tests"[Text Word]

OR "mental test"[Text Word] OR "test mental"[Text Word] OR "executive functions"[Text Word] OR "function executive"[Text Word] OR "functions executive"[Text Word] OR "executive control"[Text Word] OR "executive controls"[Text Word] OR "neuropsychological testing"[Text Word] OR "testing neuropsychological"[Text Word] OR "tests neuropsychological"[Text Word] OR "neuropsychologic tests"[Text Word] OR "neuropsychologic test"[Text Word] OR "neuropsychological test"[Text Word] OR "test neuropsychological"[Text Word] OR "cognitive testing"[Text Word] OR "testing cognitive"[Text Word] OR "cognitive tests"[Text Word] OR "cognitive test"[Text Word] OR "test cognitive"[Text Word] OR "tests cognitive"[Text Word]

(4) (1) AND (2) AND (3)

# Table S3

*Newcastle-Ottawa Scale (Normal weight vs. Obesity)*

|  | Selection | Comparability | Outcome | Total score |
| --- | --- | --- | --- | --- |
| Akın et al. (2017) | 4 | 2 | 2 | 8 |
| Alatorre-Cruz et al. (2021) | 4 | 2 | 2 | 8 |
| Blanco-Gómez et al. (2015) | 4 | 2 | 2 | 8 |
| Bozkurt et al. (2017) | 3 | 1 | 3 | 7 |
| Chojnacki et al. (2018) | 3 | 2 | 3 | 8 |
| Choudhry et al. (2013) | 4 | 2 | 2 | 8 |
| Fields et al. (2013) | 3 | 2 | 3 | 8 |
| Kamijo, Khan, et al. (2012) | 4 | 2 | 3 | 9 |
| Kamijo et al. (2014) | 4 | 2 | 3 | 9 |
| Kittel et al. (2017) | 4 | 2 | 3 | 9 |
| Krombholz (2013) | 4 | 2 | 3 | 9 |
| Li et al. (2008) | 4 | 2 | 3 | 9 |
| Logan et al. (2021) | 4 | 2 | 3 | 9 |
| Maayan et al. (2011) | 4 | 2 | 3 | 9 |
| Mayer et al. (2022) | 4 | 2 | 3 | 9 |
| Meo et al. (2019) | 4 | 1 | 3 | 8 |
| Özer et al. (2024) | 4 | 2 | 3 | 9 |
| Parhoon et al. (2023) | 3 | 2 | 3 | 8 |
| Parisi et al. (2010) | 4 | 2 | 3 | 9 |
| Pearce, Mackey, et al. (2018) | 3 | 2 | 3 | 8 |
| Qavam et al. (2015) | 4 | 2 | 2 | 8 |
| Yau et al. (2014) | 4 | 2 | 3 | 9 |
